# LAP2alpha facilitates myogenic gene expression by preventing nucleoplasmic lamin A/C from spreading to active chromatin regions

**DOI:** 10.1093/nar/gkae752

**Published:** 2024-09-04

**Authors:** Simona Ferraioli, Fatih Sarigol, Celine Prakash, Daria Filipczak, Roland Foisner, Nana Naetar

**Affiliations:** Max Perutz Labs, Vienna Biocenter Campus (VBC), Dr.-Bohr-Gasse 9 / Vienna Biocenter 5, 1030 Vienna, Austria; Medical University of Vienna, Max Perutz Labs, Dr.-Bohr-Gasse 9 / Vienna Biocenter 5, 1030 Vienna, Austria; Max Perutz Labs, Vienna Biocenter Campus (VBC), Dr.-Bohr-Gasse 9 / Vienna Biocenter 5, 1030 Vienna, Austria; Medical University of Vienna, Max Perutz Labs, Dr.-Bohr-Gasse 9 / Vienna Biocenter 5, 1030 Vienna, Austria; Max Perutz Labs, Vienna Biocenter Campus (VBC), Dr.-Bohr-Gasse 9 / Vienna Biocenter 5, 1030 Vienna, Austria; Center for Integrative Bioinformatics Vienna, University of Vienna, Dr.-Bohr-Gasse 9, 1030 Vienna, Austria; Max Perutz Labs, Vienna Biocenter Campus (VBC), Dr.-Bohr-Gasse 9 / Vienna Biocenter 5, 1030 Vienna, Austria; Medical University of Vienna, Max Perutz Labs, Dr.-Bohr-Gasse 9 / Vienna Biocenter 5, 1030 Vienna, Austria; Vienna BioCenter PhD Program, a Doctoral School of the University of Vienna and Medical University of Vienna, A-1030 Vienna, Austria; Max Perutz Labs, Vienna Biocenter Campus (VBC), Dr.-Bohr-Gasse 9 / Vienna Biocenter 5, 1030 Vienna, Austria; Medical University of Vienna, Max Perutz Labs, Dr.-Bohr-Gasse 9 / Vienna Biocenter 5, 1030 Vienna, Austria; Max Perutz Labs, Vienna Biocenter Campus (VBC), Dr.-Bohr-Gasse 9 / Vienna Biocenter 5, 1030 Vienna, Austria; Medical University of Vienna, Max Perutz Labs, Dr.-Bohr-Gasse 9 / Vienna Biocenter 5, 1030 Vienna, Austria

## Abstract

A-type lamins form a filamentous meshwork beneath the nuclear membrane that anchors large heterochromatic genomic regions at the nuclear periphery. A-type lamins also exist as a dynamic, non-filamentous pool in the nuclear interior, where they interact with lamin-associated polypeptide 2 alpha (LAP2α). Both proteins associate with largely overlapping euchromatic genomic regions in the nucleoplasm, but the functional significance of this interaction is poorly understood. Here, we report that LAP2α relocates towards regions containing myogenic genes in the early stages of muscle differentiation, possibly facilitating efficient gene regulation, while lamins A and C mostly associate with genomic regions away from these genes. Strikingly, upon depletion of LAP2α, A-type lamins spread across active chromatin and accumulate at regions of active H3K27ac and H3K4me3 histone marks in the vicinity of myogenic genes whose expression is impaired in the absence of LAP2α. Reorganization of A-type lamins on chromatin is accompanied by depletion of the active chromatin mark H3K27ac and a significantly impaired myogenic differentiation. Thus, the interplay of LAP2α and A-type lamins is crucial for proper positioning of intranuclear lamin A/C on chromatin to allow efficient myogenic differentiation.

## Graphical Abstract

**Graphical Abstract:**
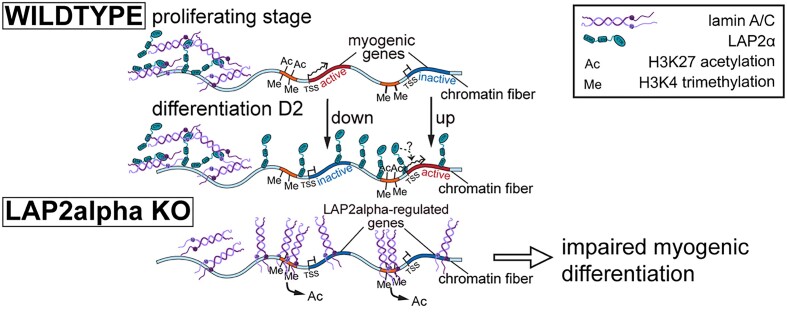

## Introduction

The nuclear lamina is a prominent architectural element in the nucleus of higher eukaryotes that is involved in chromatin organization and gene regulation. It anchors long heterochromatic regions, called lamina-associated-domains (LADs), at the nuclear periphery (1) and contributes to tissue-specific gene repression (2–4). The lamina is a scaffold structure consisting of 3.5 nm thick intermediate filaments (5) formed by lamins at the nuclear periphery. Lamins are classified as either A or B-type, based on their expression patterns and biochemical properties (6). B-type lamins are abundantly expressed in all cell types throughout development, whereas A-type lamins are expressed at low levels in embryonic stem cells but are upregulated during differentiation (7,8). Unlike B-type lamins, which remain permanently farnesylated and carboxymethylated at their C-terminus and are thus tightly associated with the nuclear membrane, lamin A undergoes an additional post-translational proteolytic cleavage step, removing the farnesylated C-terminus (6). Consequently, lamin A is less tightly bound to the inner nuclear membrane and a fraction of it can also be found in the nuclear interior (9), together with its smaller splice variant lamin C lacking the farnesylation motif altogether. Thus, A-type lamins are found in two pools: one pool at the nuclear lamina forming stable filaments, and a second pool localizing throughout the nuclear interior as a soluble, highly dynamic complex (10).

Interestingly, unlike peripheral lamins that interact mainly with heterochromatic LADs, nucleoplasmic lamins A and C can associate with large euchromatic genomic regions outside of LADs together with the nucleoplasmic LAP2 isoform, LAP2α (10,11). LAP2α is one of six splice variants encoded by the mammalian *Lap2/Tmpo* gene. It lacks a transmembrane domain found in the other isoforms, which are integrated in the inner nuclear membrane (12). LAP2α localizes throughout the nucleus, where it binds to lamins A and C and maintains the dynamic nucleoplasmic lamin pool, probably by impairing lamin A/C filament assembly (9,13). The intranuclear LAP2α-lamin A/C complex was found to function during early steps of progenitor and stem cell differentiation in several tissues (13,14). In particular, it was proposed that the LAP2α-lamin A/C complex regulates the transition of tissue progenitor cells from the quiescent state to the proliferating and/or differentiating state (13–16). Accordingly, absence of LAP2α in mice led to a delayed differentiation of muscle cells and other progenitor cell types in highly regenerative tissues (13,14).

Since cellular differentiation requires extensive gene regulation, we hypothesized that the broad interaction of LAP2α and nucleoplasmic lamins with active, gene-rich euchromatic regions might contribute to gene regulation in the early stages of differentiation. However, the mechanism of how the binding of LAP2α and lamin A/C to chromatin can affect gene expression remains elusive. Lamins were shown to influence epigenetics and gene expression via polycomb repressive complex 2 (PRC2) (17–19), via interaction with promoters and enhancers (20–22) or globally by affecting chromatin states (10,11), but their contributions to gene expression during differentiation remain unknown.

Here we address these important open questions systematically and investigate how the broad association of LAP2α and nucleoplasmic lamin A/C with euchromatin regulates early myogenic differentiation. We used muscle cells as a model system, as both lamins and LAP2α were previously shown to affect skeletal muscle differentiation *in vitro* and *in vivo* (14,17,19), and striated muscle laminopathies are among the most frequent diseases associated with mutations in *LMNA* in humans (23,24). We found that unlike lamin A/C, LAP2α translocated to genomic regions containing genes that were up- or downregulated during early stages of myoblast differentiation. Depletion of LAP2α led to a significantly impaired muscle differentiation, concomitant with altered expression of a subgroup of myogenic genes. Strikingly, upon depletion of LAP2α, lamin A/C spread along active chromatin and accumulated at regions of active H3K27ac and H3K4me3 histone marks close to genes deregulated in the absence of LAP2α. The reorganization of lamin A/C on chromatin was accompanied by depletion of the active H3K27ac histone mark. Overall, our data suggest a mechanism of gene regulation, where LAP2α is required for proper positioning of nucleoplasmic lamins on chromatin to prevent their aberrant spreading to regulatory elements of myogenic genes.

## Materials and methods

### Generation and cultivation of cell lines

Immortalized myoblasts derived from a p53 knockout mouse model (25) were maintained at 37°C and 5% CO_2_ in Ham‘s F-10 nutrient mix (GibcoTM) supplemented with 20% fetal calf serum (FCS), 100 U/ml penicillin and 100 μg/ml streptomycin (all from Sigma-Aldrich). Proliferating myoblasts were kept at <70% confluency.

Myoblasts were cultivated on collagen-coated plates at all stages of differentiation. For coating, plates were incubated in coating solution (500 μl of rat tail collagen I from Corning in 50 ml of sterile water with 57.5 μl glacial acetic acid) for at least 30 min at 37°C. The collagen solution was subsequently removed, and the plates were rinsed in PBS before seeding the cells. To induce differentiation, myoblasts were grown to 80–90% confluency (defined as day 0 of differentiation), followed by addition of low serum differentiation medium (Ham's F-10 with 5% fetal calf serum, 100 U/ml penicillin and 100 μg/ml streptomycin). During differentiation, the medium was replaced every 24 h.

To generate LAP2α knockout myoblasts, immortalized p53 knockout myoblasts were transfected at 40–50% confluency using 30 μl of polyethylenimine (PEI) and 10–15 μg of the plasmid pSpCas9(BB)-2A-GFP (pX458; plasmid #48138 from Addgene) carrying a m*Lap2*α-specific sgRNA (5′-CAAGAAAGTGAAGTCCGCTA-3′) or an empty vector as a control. Myoblasts were incubated overnight with the transfection reagents and the medium was replaced after 8–10 h with fresh growth medium. After 24 h, cells carrying the LAP2α-specific sgRNA, as well as wildtype control cells carrying the empty vector were sorted for EGFP expression using a FACS Aria Illu (Becton Dickinson), cultivated for another 36–48 hours, and then sorted again as before. Doubly bulk-sorted cells were further analyzed for efficient genome editing by sequencing of a PCR product derived from isolated genomic DNA spanning the expected Cas9 cut site (primers m*Lap2*α-f and -r; see ‘Primers’). Sequences were analyzed using the TIDE software available online (https://tide.nki.nl/) (26). Absence of LAP2α on the protein level was verified by Western blotting.

To reintroduce LAP2α protein, LAP2α knockout or wildtype myoblasts were transduced with the lentiviral vector pLVX-mCherry encoding FLAG-tagged full-length wildtype LAP2α (27) or an empty vector as a control, followed by bulk sorting for mCherry-positive cells using a FACS Aria Illu. LAP2α expression was verified by Western blotting.

To generate *Lmna* KO myoblasts, immortalized p53 knockout myoblasts were electroporated with 150 pmol recombinant Cas9 (Horizon Discovery) and a total of 400 pmol synthetic sgRNAs targeting the mouse *Lmna* gene (133.3 pmol each of sgRNA SG-040758-01, SG-040758-02 and SG-040758-03; all from Horizon Discovery) using the Amaxa Nucleofector II system with the Cell Line Nucleofector V kit (both from Lonza Bioscience) and the B-32 program. Absence of lamin A/C was verified by Western blotting.

### Microscopy

Phase-contrast images were acquired using an Axiovert 40C phase-contrast microscope (Carl Zeiss) equipped with a Canon Power Shot G12 digital camera at 100× magnification.

### Immunoblotting

Protein samples were directly lysed in Laemmli buffer and run on 10 or 12% polyacrylamide gels at 25 mA/gel. The proteins were then transferred to a 0.2 μm PVDF membrane (Thermo Scientific) at 80 V for 2 h. The membranes were blocked in 5% milk powder (Carl Roth) in PBS for 1 h at room temperature. After a 5 min wash in PBS, the membranes were incubated with the primary antibody solution (primary antibody diluted in PBS containing 2% bovine serum albumin from Sigma-Aldrich and 0.02% NaN_3_) at 4°C overnight (see section ‘antibodies’ below). The membranes were then washed 3 times for 5 min in PBST (0.05% Tween-20 in PBS) and incubated 2 h at room temperature with the secondary antibody dilution. For signal detection, the membranes were briefly washed in PBS and the signal was detected using PierceTM ECL or ECL-plus Western blotting substrates and visualized with a Bio-Rad ChemiDocTM.

### RNA-sequencing (RNA-seq)

Proliferating myoblasts were seeded on collagen-coated plates and either harvested at <70% confluency (proliferating state) or after reaching 80–90% confluency (D0; day 0 – start of differentiation). Confluent cells were induced to differentiate by switching to low serum medium (5% FCS) and harvested after 48 h (D2; day 2 of differentiation). Total RNA was extracted using the RNAeasy® Mini kit (Qiagen), according to manufacturer's instructions.

Total RNA was submitted to the Next Generation Sequencing facility at the Vienna Biocenter Core facilities (VBCF; https://www.viennabiocenter.org/vbcf/next-generation-sequencing/), Vienna, Austria for library preparation, which included polyA mRNA enrichment with the NEBNext® Poly(A) mRNA Magnetic Isolation Module, followed by library preparation using the NEBNext® UltraTM II Directional RNA Library Prep Kit for Illumina (both New England Biolabs) and sequencing on the Illumina platform (Illumina HiSeq 2500) with SR50 mode (single-end reads; 50 bp length).

Unaligned bam files of the strand-specific 50bp single-end read sequencing libraries were converted to fastq format with bam2fastq v1.1.0 from BEDTools (28) and reads were trimmed with cutadapt v1.12 (https://github.com/marcelm/cutadapt) for both, truseq adapters and low-quality bases below Q20 at the end of the read. Trimmed reads shorter than 20 bases were discarded. The reads were then mapped with NextGenMap 0.5.2 (29) to the *Mus musculus* GRCm38.86 transcriptome reference that was prepared using RSEM v1.2.19 (30) rsem-prepare-reference, performing an ungapped end-to-end alignment (parameters: –end-to-end –gap-read-penalty 2000 –gap-ref-penalty 2000 –gap-extend-penalty 2000). Additional non-default parameters were –min-residues 1 (only full-length mapped reads were reported) and –strata -n 70, which permits up to 70 highest scoring mappings for any given read to be reported, allowing multiple mapping of reads to transcript isoforms of the same gene with 70 being the highest number of isoforms observed per gene in the reference annotation. A custom script was used to remove reads that mapped with an overhang to the transcript. Transcript abundance quantification was done using rsem-calculate-expression with a minimum fragment length of 5. Library-specific mean and standard deviation of fragment length values were inferred from fragment analyser plots. Alignment seed length of 13 and probability of 0 of generating a read from the forward strand of a transcript was used to indicate a strand-specific protocol, where all reads are derived from the reverse strand. Both, posterior mean estimates and 95% credibility intervals were calculated. Transcript-level estimates were imported and summarized to gene-level estimates using the R package tximport (31). To exclude genes with low expression, only genes with expected counts of more than 5 in all 3 libraries were kept. The counts were TMM normalized using the calcNormFactors function from edgeR (32) and transformed with limma-voom (33). Differential gene expression analysis was done using the limma workflow utilizing a single factor model that combines genotype and proliferation/differentiation stage. To account for a sequencing lane batch effect, a blocking factor was specified in the model fitting based on the lane of the library. Correlations between the samples for each lane were estimated with the duplicateCorrelation function. Statistical tests for the contrast of interest being equal to zero were performed with a moderated t-statistics test. Significant differentially expressed genes were defined as genes with minimum expression of 10 counts per million in at least one library in the comparison, an absolute log2 fold change larger than 1.5 and *P*-value <0.05.

Genes with a minimum Fragments Per Kilobase Million (FPKM) value of 0.5 in all three replicates were defined as ‘expressed’.

### ChIP-sequencing (ChIP-seq) and ChIP combined with quantitative PCR (ChIP-qPCR)

Myoblasts were seeded on 15 cm collagen-coated dishes in medium containing 20% FCS and either harvested the next day at less than 70% confluency (proliferating condition) or switched to 5% FCS at 80–90% confluency and harvested after an additional 48 h (differentiation day 2). For harvesting, cells were washed with PBS (Dulbecco's Phosphate Buffered Saline with CaCl_2_ and MgCl_2_; Sigma-Aldrich) and then incubated for 10 min at room temperature with 1% methanol-free formaldehyde (Thermo Scientific) in PBS (Dulbecco's phosphate buffered saline without CaCl_2_ and MgCl_2_; Sigma-Aldrich) on a shaker. Formaldehyde was quenched by adding glycine to a final concentration of 125 mM and incubating for 5 min at room temperature on a shaker. Myoblasts were washed twice in ice-cold PBS and harvested in PBS containing protease inhibitors (cOmplete EDTA-free protease inhibitor cocktail tablets, Roche) using low protein-binding 15ml tubes (Eppendorf). The collected cells were centrifuged at 500 x g for 5 min at 4°C, the pellet was resuspended in ice-cold WASH buffer 1 (10 mM HEPES, 0.25% Triton X-100, 10mM EDTA, 0.5 mM EGTA, cOmplete protease inhibitors and 0.1 mM PMSF in milli-Q water) at a concentration of 2 million cells per ml of buffer and incubated for 10 min on ice. The samples were again centrifuged at 500 x g for 5 min at 4°C and the pellet was resuspended in ice-cold WASH buffer 2 (10 mM HEPES, 200 mM NaCl, 1 mM EDTA, 0.5 mM EGTA, cOmplete Protease inhibitors and 0.1 mM PMSF in milli-Q water) at a concentration of 2 million cells per ml of buffer. The samples were immediately centrifuged again with the same settings and resuspended in lysis buffer (50 mM Tris–HCl pH 8.1, 1% SDS, 10 mM EDTA, cOmplete protease inhibitors and 0.1 mM PMSF in milli-Q water) at a concentration of 10 million cells per ml of buffer. The chromatin samples were incubated over night at 4°C on a rotor. The chromatin was sonicated in 15 ml sonication tubes containing 500 mg sonication beads and 900 μl chromatin sample per tube using the Bioruptor PICO with the Bioruptor water cooler (all from Diagenode; settings: 30 s ON/30 seconds OFF for 5 cycles). The sonication conditions were initially optimized for each genotype and differentiation stage and allowed for optimal enrichment of the euchromatic chromatin fraction (11). Optimization revealed very similar fragment size distributions for proliferating and differentiating wildtype and LAP2α knockout myoblasts (see also Supplementary Figure S3). Sheared chromatin was diluted with ChIP dilution buffer (16.72 mM Tris–HCl pH 8.1, 167.4 mM NaCl, 1.2 mM EDTA, 1.1% Triton-X 100, 0.001% SDS, cOmplete protease inhibitors) in a 2:1 ratio (sample:buffer), snap-frozen in liquid N_2_ and stored at –80°C. Before freezing, a 100 μl chromatin aliquot was set aside to confirm sufficient chromatin shearing.

For the immunoprecipitation (IP), chromatin samples were thawed on ice and centrifuged at 18 000 x g for 15 min at 4°C to remove insoluble precipitates. Chromatin concentration was determined using the Qubit broad range dsDNA Quantitation kit (Invitrogen/Thermo Scientific). 15–20 μg of chromatin was diluted with ChIP dilution buffer to a final volume of 1–1.5 ml and incubated with the appropriate antibody (for exact amounts, see section ‘antibodies’ below) overnight at 4°C. At this stage, 1 μg of chromatin was set aside as INPUT. 40 μl of pre-washed Pierce protein A/G magnetic beads (Thermo Scientific) were added to each sample and incubated 4–5 h at 4°C while rotating. The supernatant was removed and the beads were washed with 1 ml of the following buffers in this order: RIPA buffer (50 mM Tris–HCl pH 8.0, 150 mM NaCl, 0.1% SDS, 0.5% sodium deoxycholate and 1% NP-40 in milli-Q water), High-Salt buffer (50 mM Tris–HCl pH 8.0, 500 mM NaCl, 0.1% SDS and 1% NP-40 in milli-Q water), LiCl buffer (50 mM Tris–HCl pH 8.0, 250 mM LiCl, 0.5% sodium deoxycholate and 1% NP-40 in milli-Q water), and twice with TE buffer (10 mM Tris–HCl pH 8.0 and 1 mM EDTA in milli-Q water). For each washing step, beads were incubated in the wash buffer for 10 min at 4°C while rotating. After the final wash, the supernatant was removed completely and 200 μl elution buffer (100 mM NaHCO_3_, 2% SDS, 10 mM DTT) was added to each sample (and to the INPUT samples). Samples were incubated for 30 min at room temperature, shaking at 1200 RPM. From this step onwards, the INPUT samples were processed together with the IP samples. The supernatant (without beads), now containing the precipitated chromatin, was collected and chromatin was decrosslinked by adding 10 μl of 4 M NaCl per 200 μl sample and incubating the solution over night at 65°C in a shaker at 300 RPM. 4 μl of 0.5 M EDTA, 8 μl of 1 M Tris–HCl pH 6.5 and 0.5 μl RNAse A (10 mg/ml DNase and protease free RNase from Thermo Scientific) were added to each sample and samples were incubated for 1 h at 37°C in a shaker at 300 RPM. Proteinase K (Thermo Scientific) was added to a final concentration of 250 μg/ml and samples were incubated for 1–2 h at 55°C in a shaker at 300 RPM. The DNA was purified using the ChIP DNA Clean & Concentrator kit by Zymo Research and eluted in 30 μl of milli-Q water. DNA concentration was determined using the Qubit high sensitivity dsDNA Quantitation kit (Invitrogen/Thermo Scientific).

For ChIP-qPCR, purified DNA was analyzed using the KAPA SYBR Green 2x PCR master mix (Kapa Biosystems) and primers specific to fragments within the regulatory region (±1 kB up- and downstream of the transcription start site) of the genes *Cap2* and *Jph2* (*Cap2*-f: 5′-GTCACTATGCAGCCCTACCC-3′, *Cap2*-r: 5′-CAAGCAGGAAATGCCTTCGC-3′; *Jph2*-f: 5′- GAGCAAGACTCACCTCCGTC-3′, *Jph2*-r: 5′-ACAGTGGTGCCAAGTACGAG-3′). The analysis was performed using an Eppendorf Realplex 2 Mastercycler following the manufacturer's instructions. To normalize for batch-to-batch variability, the % input for each specific genomic locus was divided by the average % input of this locus across all samples of the relevant biological replicate. These values were subsequently used for statistical analyses.

For ChIP-sequencing, the DNA was delivered to the Next Generation Sequencing facility at the Vienna Biocenter Core Facilities (VBCF), which generated the library using the NEBNext® UltraTM II DNA Library Prep Kit for Illumina and sequenced the samples on an Illumina platform (HiSeq 2500 and NovaSeq 6000) with SR100 mode (single-end reads; 100 bp length).

Raw sequencing reads from ChIP-seq experiments in bam format were extracted using bamtofastq from bedtools. The quality of raw sequencing reads was checked using FastQC (https://www.bioinformatics.babraham.ac.uk/projects/fastqc/). We mapped the raw sequencing reads to the *Mus musculus* genome assembly GRCm38 (mm10) using NextGenMap version 0.5.2 (29) using default settings. Mapping statistics were evaluated using samtools (34). For lamin A/C and LAP2α ChIP-seq experiments, peaks were called using the enhanced domain detector (EDD) software (35) with default parameters. Peaks for H3K27ac and H3K4me3 ChIP-seq were called using MACS2 (36) (parameters H3K27ac: -q 0.0005 -m 5 50 –keep-dup 1; parameters H3K4me3: -q 0.005 -m 5 50 –keep-dup 1). We used bamCompare from deepTools (37) to generate log2 ratio files, which were used for visualization using the Integrative Genomics Viewer (IGV) (38). Coverage files of the mapped reads were generated using bamCoverage from deepTools with the RPKM normalization method. When needed, file formats were converted using bigWigToBedGraph and bedGraphToBigWig (39). The correlation between ChIP-seq replicates was calculated using multiBamSummary bins from deepTools.

We used an in-house written script in R (https://www.R-project.org/) to calculate and illustrate the means and medians of log2 ratio signals across genomic regions of interest. The log2 ratio and RPKM coverage values were also illustrated as heatmaps with mean signals using computeMatrix and plotHeatmap from deepTools.

### Intersections and overlaps

Overlapping peak coordinates between samples of interest were found using bedtools intersect –wao, followed by calculation of the total lengths of overlapping and non-overlapping genomic bases, which were plotted as Venn diagrams using meta-chart (https://www.meta-chart.com/).

Genes that overlap with lamina-associated domains (LADs) were identified using bedtools intersect command on genomic coordinates of genes and LAD regions with a minimum of 1bp overlap. LAD regions were obtained from the NCBI GEO database (ID: GSE17051) (40) and converted for the mm10 genome assembly version.

Closest distances between genomic regions of interest were calculated using closest-features –dist from BEDOPS (41), taking both chromosomal directions into account, and were summarized in bins of distances.

To define a gene as bound by a protein of interest, the gene was subdivided into 10 bp windows, calculating the ChIP-seq log2 ratio of the respective protein for each window. The average binding value was obtained by summing up the values of the individual windows and dividing them by the number of windows analyzed. For further analyses, we arbitrarily defined the top 10% of genes ranked according to their binding values as bound (lowest average log2 ratio value for bound genes: 0.35–0.87, depending on the specific antibody).

### Functional analysis

Gene set enrichment analysis was performed with enrichGO function of clusterProfiler v3.18.1 (Bioconductor package) and the results were illustrated using the dotplot function in R.

To find datasets in the Cistrome database that are most similar to our ChIP-seq peak sets or any other region of interest, we used the CISTROME DB Toolkit (42), which uses the GIGGLE search engine (43) identifying and ranking the significance of genomic loci shared between genome intervals.

### Antibodies

The following antibodies were used for ChIP-seq analyses: anti-LAP2α 1H11 (300 μl undiluted hybridoma supernatant per ChIP sample; Max Perutz Labs Monoclonal Antibody facility) (11); anti-lamin A/C 3A6 (300 μl undiluted hybridoma supernatant per ChIP sample; Max Perutz Labs Monoclonal Antibody facility) (11); anti-lamin A/C E1 (10 μl per ChIP sample; Santa Cruz Biotechnology, Cat#sc-376248); anti-H3K27ac (20 μl per ChIP sample; Merck/Millipore, Cat#07-360); anti-H3K4me3 (10 μl per ChIP sample; Merck/Millipore, Cat#07-473) and anti-lamin B1 (4 μl per ChIP sample; Proteintech, Cat#12987-1-AP).

Antibodies used for ChIP-qPCR analyses were: anti-lamin A/C 3A6 (300 μl undiluted hybridoma supernatant per ChIP sample); anti-H3K27ac (20 μl per ChIP sample; Merck/Millipore, Cat#07-360) and anti-HDAC1 10E2 (300 μl undiluted hybridoma supernatant per ChIP sample; Max Perutz Labs Monoclonal Antibody facility).

For Western blotting the following antibodies were used: anti-LAP2α 1H11 (dilution 1:500) and anti-lamin A/C 3A6 (dilution: 1:500) (11); anti-Myogenin (dilution 1:10; F5D supernatant, Developmental Studies Hybridoma Bank DSHB); anti-myosin heavy chain (MyHC) (1:10 dilution; MF-20 supernatant, DSHB); anti-γ-tubulin (1:5000 dilution; clone GTU88, Sigma-Aldrich, Cat# T6557) and anti-LAP2 Ab12 (undiluted) (44).

### Primers and TIDE-PCR

Primers used for PCR to perform TIDE analysis were m*Lap2*α-f (5′-GGGTCTTTTATGGGCCATTTTTGT-3′) and m*Lap2*α-r (5′- CTCTTCCCTCCACGGCAAA-3′). PCR was performed at an annealing temperature of 67°C using the Q5 high fidelity DNA polymerase (New England Biolabs) according to manufacturer's instructions. 10% betaine (Sigma-Alrich) was added to each PCR reaction to aid amplification of GC-rich sequences. The primer m*Lap2*α-f was used for sequencing the PCR products.

### Statistical analysis

To test the statistical significance of the overlap between a set of genes and a ChIP-seq peak set of interest, we calculated the amount of expected overlaps from randomly selected regions in the genome of the same lengths as the specific peak set using the overlapPermTest function of regioneR R package (45), which performs a permutation test by providing a Z-score and a *P*-value to find out whether the overlap between two sets of regions is higher or lower than expected by chance. To compare the distributions of the closest distances between specific sets of genes and ChIP-seq peak sets, we used the Kolmogorov–Smirnov test (KS test) calculated in R Statistical Software (v4.0.3; R Core Team 2020) using dgof package. To correct for multiple testing, we used p.adjust function in R with the Hochberg method to correct the *P*-values of all relevant KS tests. To compare ChIP-qPCR results for wildtype and LAP2α knockout cells, two-tailed, two-sample, equal variance student's t-tests were performed. Statistical significance of the differential expression of genes was calculated by moderated T-tests.

## Results

### LAP2α knockout myoblasts display deregulation of a subset of myogenic genes and impaired differentiation

**Figure 1.**
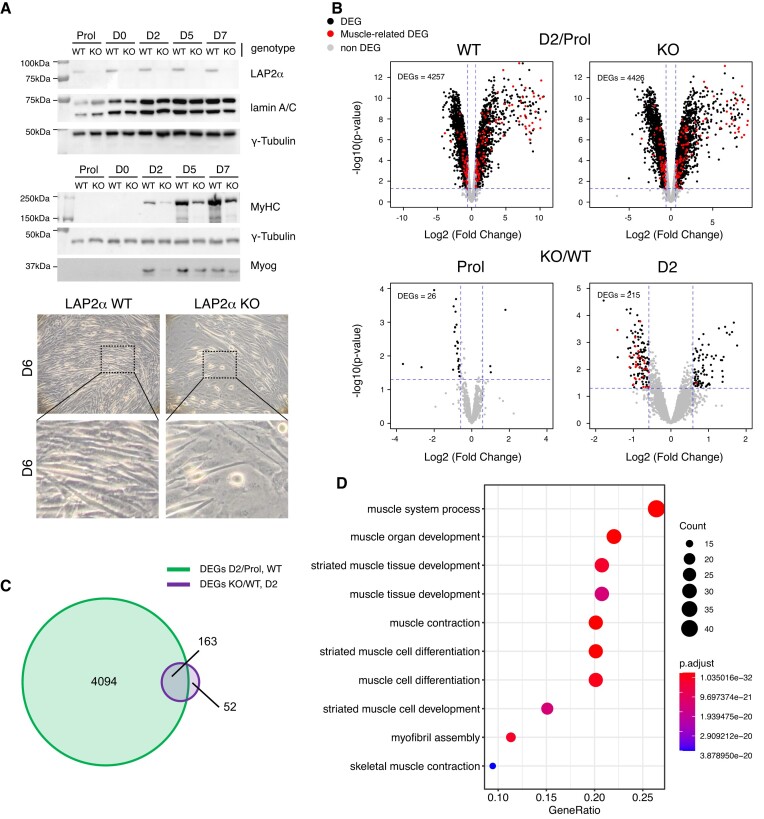
Depletion of LAP2α in myoblasts causes impaired differentiation affecting a subgroup of myogenic genes in early differentiation stages. (**A**) Wildtype (WT) and LAP2α knockout (KO) immortalized myoblasts were differentiated *in vitro* for 7 days (D0–D7) or kept in the proliferating stage (Prol) and analyzed by Western blotting (upper panel) for the expression of LAP2α, lamin A/C and different myogenic markers as indicated on the right (MyHC: Myosin heavy chain; Myog: Myogenin). Images of wildtype and LAP2α knockout cells were obtained by phase-contrast light microscopy (100× magnification) six days after the induction of differentiation (lower panel). The dashed box denotes the area shown as enlarged image below. (**B**) Wildtype and LAP2α knockout cells were differentiated as in (A), RNA was isolated and analyzed by RNA-sequencing. Volcano plots display differentially expressed genes (DEGs) in wildtype and knockout differentiating (D2: day 2 of differentiation) versus proliferating (Prol) myoblasts (upper panel). DEGs in LAP2α knockout versus wildtype proliferating and differentiating cells (D2) were also analyzed (lower panel). Significantly differentially expressed genes are depicted in black. Genes related to muscle differentiation are depicted in red. Non-significantly changed genes are depicted in grey. (**C**) Venn diagram displaying the overlap of differentially expressed genes in differentiating (D2) versus proliferating wildtype myoblasts (DEGs D2/Prol WT; 4257 genes) and differentiating (D2) LAP2α knockout versus wildtype myoblasts (DEGs KO/WT D2; 215 genes). (**D**) 163 overlapping genes from (**C**) were subjected to gene-ontology (GO) analysis. Most significantly-enriched GO terms for biological processes are depicted, including the number of genes (count) found in each specific GO term, the fraction of genes compared to all genes within that GO term (gene ratio) and the adjusted *P* value (*P* adjust).

To study the role of LAP2α in myogenic differentiation, we disrupted the *Lap2α* gene in an immortalized myoblast cell line derived from a p53 knockout mouse model (25) using a CRISPR-Cas9 approach (Supplementary Figure S1). To account for any unspecific changes caused by the expression of Cas9, wildtype control myoblasts were treated in the same way as LAP2α knockout cells but using the Cas9 plasmid lacking the LAP2α-specific sgRNA. We used immortalized, p53-deficient myoblasts, as primary myoblasts have limited proliferation capacity *in vitro* and are often contaminated with fibroblasts making them unsuitable for experiments performed in bulk. In addition, the p53-deficient myoblasts were shown to differentiate into fully functional contractile myotubes (25), while spontaneously immortalized myoblasts, such as the commonly used C2C12 cell line, have limited myogenic potential (46). LAP2α was efficiently depleted by Cas9-mediated knockout in myoblasts as confirmed by Western blotting (Figure 1A), whereas the expression of the other major LAP2 isoform, LAP2β, was unaffected (Supplementary Figure S1C). LAP2α knockout and wildtype control cells were differentiated into myotubes for 7 days. In accordance with previous findings (14), LAP2α-depleted myoblasts showed a significantly impaired myogenic differentiation with reduced expression of the differentiation markers myosin heavy chain (MyHC) and Myogenin, and reduced formation of mature myotubes compared to wildtype controls (Figure 1A).

To investigate the changes associated with this impaired differentiation on a genome-wide level, we performed RNA-seq analysis of proliferating and differentiating LAP2α knockout and wildtype myoblasts. Since previous studies suggested a function of LAP2α mainly in early differentiation stages (13,14), we analyzed gene expression changes at the earliest stage of muscle differentiation, when cells are first becoming confluent (referred to as D0 of differentiation), and at day 2 of differentiation (D2) following serum deprivation (Supplementary Figure S2A). Whereas only minor gene expression changes were observed in D0 cells when compared to proliferating myoblasts (∼50 differentially expressed genes; Supplementary Figure S2B), >4000 genes were differentially expressed as early as 2 days after induction of differentiation in both, LAP2α knockout and wildtype control cells (Figure 1B; Supplementary Table S1). We therefore compared gene expression profiles of LAP2α knockout and wildtype cells in the proliferating state and at differentiation day 2, when major changes in gene expression take place. Interestingly, only 26 genes were differentially expressed in proliferating LAP2α knockout compared to wildtype myoblasts (Figure 1B, lower left panel; Supplementary Table S2). However, 215 genes showed differential expression in the absence of LAP2α after 2 days of differentiation (Figure 1B, lower right panel; Supplementary Table S3). Among these genes, with approx. 2/3 being downregulated, were several muscle-related genes (Figure 1B, myogenic genes marked in red). Intriguingly, the large majority of genes deregulated in LAP2α knockout cells (163 out of 215 Differentially Expressed Genes; DEGs KO/WT D2) overlapped with genes differentially expressed during early stages of muscle differentiation in wildtype cells (DEGs D2/Prol WT; Figure 1C). Gene ontology (GO) analysis of these 163 overlapping genes revealed a highly significant enrichment of genes related to muscle differentiation, function, and development (Figure 1D). Notably, when we compared publicly available transcription factor ChIP-seq data sets (from the CISTROME database) (47) with the regulatory regions of genes found in the top 10 GO terms as shown in Figure 1D, several known major transcriptional regulators of myogenesis, including Myogenin, MEF2 and MyoD came up as top hits (Supplementary Figure S2C). Thus, depletion of LAP2α in muscle cells leads to deregulation of a subset of differentiation-induced myogenic genes, concomitant with a compromised differentiation of LAP2α knockout versus wildtype myoblasts.

### LAP2α, but not lamin A/C moves towards genomic regions containing myogenic genes in differentiating myoblasts

**Figure 2.**
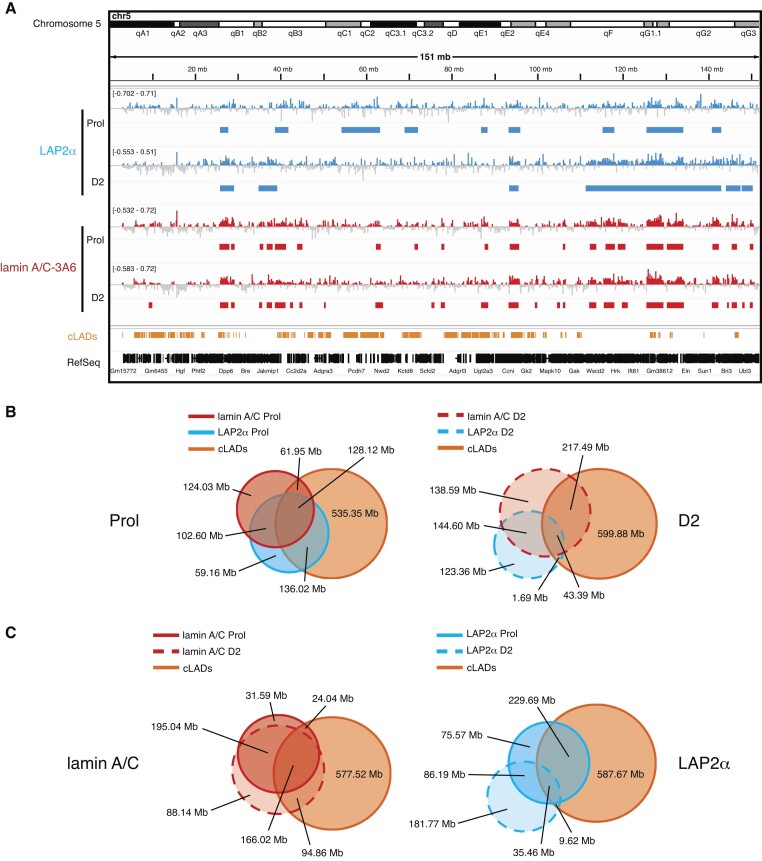
LAP2α and lamin A/C bind to large overlapping euchromatic regions in myoblasts and partially relocate during early myogenic differentiation. (**A**) ChIP-seq analysis was performed in proliferating (Prol) and differentiating wildtype immortalized myoblasts (D2: day 2 of differentiation) for LAP2α (blue) and lamin A/C (3A6 antibody; red) as indicated. IGV browser was used to display log2 ratio of ChIP over input signal tracks of mouse chromosome 5. Positive log2 ratio values are depicted in color, negative values in grey. Peaks called by the Enriched Domain Detector software (EDD) are depicted for each ChIP track. The scale of each log2 ratio track is indicated on the left. cLADs: constant lamina-associated domains. RefSeq: Gene annotations are from the NCBI reference sequence database. (**B**) Venn diagrams depicting the overlap of LAP2α (blue circle) and lamin A/C ChIP EDD peaks (red circle) in proliferating cells (left panel, solid lines) and differentiating cells (D2; right panel, dashed lines). Additionally, the overlap with cLAD regions is displayed (orange circle). The total genomic lengths of overlapping and non-overlapping regions between peak sets were identified using the intersect function of the BEDTools suite and are shown in megabases (Mb). (**C**) Venn diagrams as in (B) but depicting the overlap of lamin A/C (red circles, left panel) and LAP2α (blue circles, right panel) EDD peaks in proliferating (solid lines) and differentiating cells (D2; dashed lines).

In order to test if and how interactions of LAP2α and lamin A/C with chromatin might impact myogenic gene expression, we performed ChIP-seq of LAP2α and lamin A/C in proliferating and differentiating myoblasts employing mild sonication conditions, allowing for the enrichment of euchromatic chromatin fractions (11). Sonication under these conditions generated similar fragment size distributions for proliferating and differentiating wildtype and LAP2α knockout samples (Supplementary Figure S3). We then called ChIP-seq peaks using the Enriched Domain Detector (EDD) software, suitable for the identification of broad enrichment domains (35) (Figure 2). In previous studies, LAP2α and lamin A/C were shown to bind to large (1–2 Mb) overlapping euchromatic genomic domains in fibroblasts when applying this experimental approach (11). Similarly, in proliferating myoblasts and at differentiation day 2, LAP2α and lamin A/C EDD peaks covered 11–20% of the genome with an average peak length of 1- 3 Mb (Supplementary Figure S4A). Genomic sites bound by LAP2α and lamin A/C overlapped by 55% in proliferating myoblasts and by 35% at differentiation day 2 (Figure 2B). Lamin A/C- and LAP2α-bound genomic regions also overlapped with heterochromatic constitutive LADs (cLADs; i.e. LADs that are constitutively associated with the lamina in different cell types) (40,48) to a varying extent depending on the differentiation stage (LAP2α Prol: 62%; LAP2α D2: 14%; lamin A/C Prol: 46%; lamin A/C D2: 48%; Figure 2B). Importantly, these data show that at least 40% of LAP2α and lamin A/C binding sites identified by ChIP-seq are located in active euchromatic regions outside of cLADs. Interestingly, while lamin A/C showed only a modest relocalization on chromatin in early stages of muscle differentiation (Figure 2C, left panel), LAP2α relocated more substantially, moving from more heterochromatic, LAD-overlapping regions in proliferating cells to euchromatic regions in D2 myoblasts (Figure 2C, right panel). Lamin A/C ChIP-seq using two different lamin A/C antibodies directed to a C- or N-terminal region of lamin A/C (antibody 3A6 versus E1) (11) revealed similar results showing 70% - 80% overlapping regions (Supplementary Figure S4B and C). We thus used mostly the antibody to the C-terminus of lamin A/C (3A6) for further analyses. Altogether, LAP2α and lamin A/C ChIP-seq analyses revealed binding of these proteins to overlapping genomic regions within both, hetero- and euchromatin in myoblasts and demonstrated that both proteins partially relocate on chromatin during early stages of myogenic differentiation.

**Figure 3.**
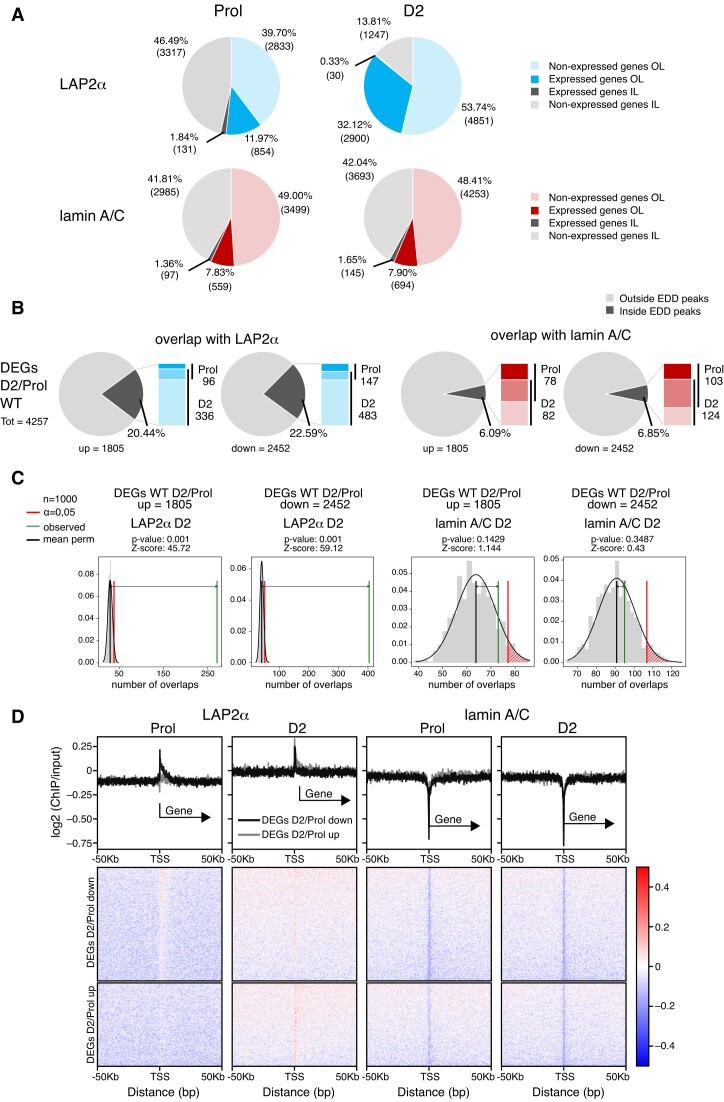
LAP2α relocalizes to chromatin regions containing myogenic genes in differentiating cells. (**A**) Pie charts depicting the percentages of expressed and non-expressed genes within LAP2α (blue; upper panel) or lamin A/C (red; lower panel) EDD peaks in proliferating (Prol; left panel) and differentiating cells (D2; right panel), additionally distinguishing genes outside of cLADs (OL; in color) and inside of cLADs (IL; in grey). Numbers in parentheses correspond to absolute numbers of genes. (**B**) Pie charts showing the percentages of differentially expressed genes during early differentiation of wildtype cells (DEGs D2/Prol WT; 4257 genes) split into up- (1805) and downregulated (2452) genes that are found within or outside of LAP2α (panel 1 and 2) and lamin A/C (panel 3 and 4) EDD peaks. Bar charts on the right of each pie chart depict the number of DEGs found within EDD peaks in each differentiation stage (proliferating versus D2; dark versus light color, respectively; genes found in both stages are depicted in an intermediate color). (**C**) EDD peaks for LAP2α (panel 1 and 2) and lamin A/C (panel 3 and 4) in differentiation stage D2 were randomized 1000 times to yield genomic regions of the same size and the overlap with up- and downregulated DEGs D2/Prol WT was determined. The distributions of these randomized overlaps were plotted as histograms with the red line demarcating statistical significance at α=0.05 and the green line marking the observed overlap of DEGs with EDD peaks. Mean perm: mean value for random permutation tests (black line). (**D**) Heat maps displaying log2 ratio signal (ChIP over input) for LAP2α (panels 1 and 2) and lamin A/C (panels 3 and 4) in proliferating (Prol) and differentiating (D2) myoblasts on genes that are downregulated (DEGs D2/Prol down) or upregulated (DEGs D2/Prol up) during early differentiation. Graphs on top of heatmaps show mean log2 ratio tracks on down- (black) or upregulated genes (grey). TSS: transcription start site.

To better understand how LAP2α and lamin A/C binding to chromatin might influence gene expression during myogenic differentiation, we first determined whether genes found within LAP2α or lamin A/C EDD peaks within or outside of cLADs are expressed or non-expressed (Figure 3A). Whereas ∼90% of lamin-overlapping genes were not expressed, both inside and outside of cLADs in proliferating and D2 myoblasts (Figure 3A, lower panel), the fraction of expressed genes in LAP2α-bound regions increased from ∼14% in proliferating cells to ∼32% in differentiating myoblasts (mostly outside cLADs; Figure 3A, upper panel). Thus, LAP2α, but not lamin A/C relocates to euchromatic genomic regions containing a significant number of expressed genes in early stages of differentiation.

We then tested whether lamin A/C- or LAP2α-bound genomic regions contained also genes whose expression changes in early stages of muscle differentiation. While only a small minority (∼6%) of the 4257 differentially expressed genes (DEGs D2/Prol) overlapped with lamin-bound regions, more than 20% of these myogenic DEGs were located within LAP2α EDD peaks primarily in D2 cells (Figure 3B), independent whether they were up- or downregulated in early stages of differentiation. This number was much higher than expected by chance, as randomized genomic regions with the same size as LAP2α EDD peaks showed a significantly lower overlap with the up- and downregulated genes (random permutation testing; Z-score_upregulated_ = 45.72, Z-score_downregulated_ = 59.12, *P*-value = 0.001; Figure 3C). In contrast, the number of genes located in lamin A/C-bound regions was similar to that in randomized genomic regions (Figure 3C).

To investigate whether LAP2α or lamin A/C bind directly to genes differentially expressed in differentiation, we compiled heat maps of LAP2α or lamin A/C ChIP log2 signal (ChIP/input) on up- and downregulated genes (DEGs D2/Prol) (Figure 3D). In accordance with the low overlap of genes with lamin A/C EDD peaks, the transcription start site (TSS) of DEGs and their surrounding area were largely depleted of lamins in proliferating and D2 cells, with a more significant depletion directly at the TSS (Figure 3D, 3rd and 4th panel). For LAP2α we noticed a similar overall depletion on differentially expressed genes and the surrounding area in proliferating cells, whereas in cells at differentiation day 2 LAP2α showed a mild enrichment at the TSS of DEGs and within 50 kb up- and downstream of the TSS without any noticeable accumulation on gene bodies (Figure 3D, first and second panel). As transcription start sites of active genes were shown to be prone to non-specific enrichment in ChIP (49,50) and LAP2alpha enrichment on these sites is very mild, the TSS signal may be unspecific and/or physiologically not relevant. Thus, LAP2α does not significantly enrich on differentially expressed genes, but shows overall weak association with genomic regions containing these genes, particularly at differentiation state D2. Accordingly, when we determined the average ChIP log2 signal of lamins and LAP2α on individual genes, defining genes within the top 10% of signal strength as bound, very few DEGs Prol/D2 within LAP2α or lamin A/C EDD peaks came up as bound (<10%; Supplementary Figure S5A).

In summary, while lamin A/C mostly binds to genomic regions away from differentially expressed genes during differentiation, LAP2α binds to similar, lamin A/C-overlapping, gene-depleted chromatin regions in proliferating cells, but during early stages of differentiation, LAP2α relocates towards regions containing myogenic genes, without accumulating on these genes directly. Altogether, these findings suggest that LAP2α might have an active regulatory role in gene expression during early stages of muscle differentiation, in accordance with the observation that a subset of differentiation-regulated DEGs is altered in its absence (Figure 1B–D).

### Lamin A/C spreads towards deregulated genes within euchromatic regions upon LAP2α depletion

**Figure 4.**
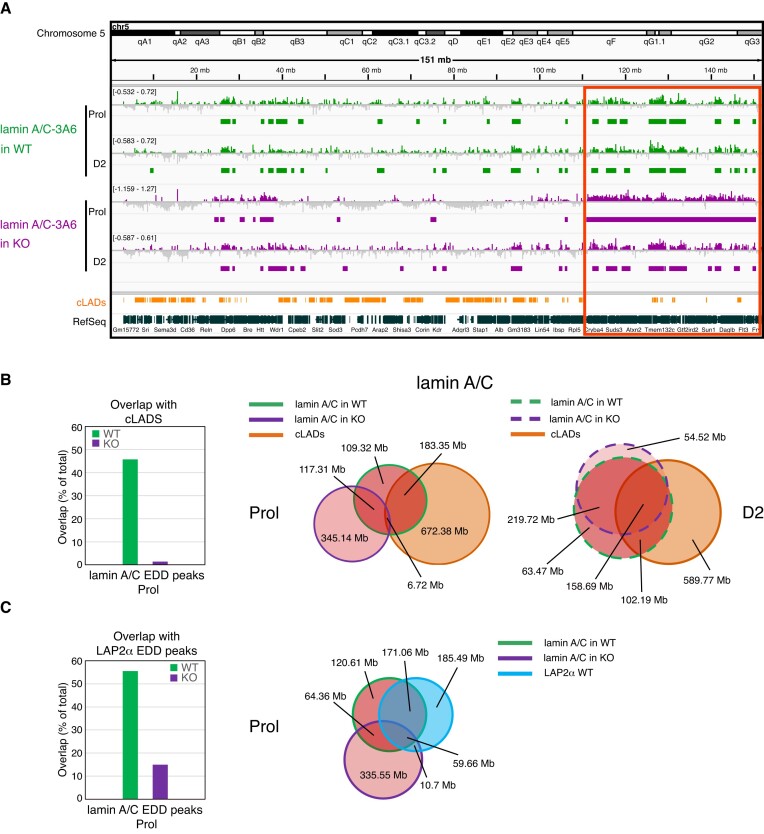
Lamin A/C spreads on euchromatin upon LAP2α depletion, moving away from cLADs and formerly LAP2α-bound regions. (**A**) ChIP-seq analysis was performed in proliferating (Prol) and differentiating (D2) wildtype (WT; green) and LAP2α knockout (KO; purple) myoblasts using lamin A/C antibody 3A6 as indicated. IGV browser was used to display log2 ratios of ChIP over input signal tracks of mouse chromosome 5. Positive log2 ratio values are depicted in color, negative values in grey. Peaks called by the Enriched Domain Detector software (EDD) are depicted for each ChIP track. The scale of each log2 ratio track is indicated on the left. Red box demarcates regions of lamin spreading on chromatin in proliferating LAP2α knockout cells. cLADs: constant lamina-associated domains. RefSeq: Gene annotations from the NCBI reference sequence database. (**B**) Venn diagrams depicting the overlap of lamin A/C ChIP EDD peaks in wildtype (red circles with green line) and LAP2α knockout (red circles with purple line) proliferating (Prol; middle panel, solid lines) and differentiating cells (D2; right panel, dashed lines). Additionally, the overlap with cLAD regions is displayed (orange circle). The total genomic lengths of overlapping and non-overlapping regions between peak sets were identified using the intersect function of the BEDTools suite and are shown in megabases (Mb). Bar graph on the left displays the overlap in % of lamin A/C EDD peaks with cLADs in wildtype and LAP2α knockout cells. (**C**) Venn diagrams as in (B), but depicting the overlap of lamin A/C ChIP EDD peaks in wildtype and LAP2α knockout proliferating cells (red circles with green and purple line, respectively) with wildtype LAP2α EDD peaks (blue circle). Bar graph on the left displays the overlap in % of lamin A/C EDD peaks in wildtype and LAP2α knockout cells with wildtype LAP2α EDD peaks.

As we previously showed that loss of LAP2α affects the properties of lamins A and C, including their mobility, assembly status and chromatin interaction (9,11), we tested genome-wide chromatin association of A-type lamins in myoblasts upon depletion of LAP2α. Excitingly, lamin A/C ChIP-seq in LAP2α-depleted myoblasts revealed a major relocalization of lamin A/C on chromatin compared to LAP2α wildtype cells, particularly in proliferating myoblasts (Figure 4). Lamin A/C-bound regions in LAP2α knockout cells showed less than 30% overlap with those in wildtype myoblasts (Venn diagram in Figure 4B, middle panel). Furthermore, lamin A/C spread towards active, cLAD-depleted, euchromatic regions in LAP2α knockout cells, clearly detectable also in ChIP tracks displayed in the IGV browser (Figure 4A, red box). Venn diagrams revealed a drastic reduction of the overlap of lamin A/C-bound sites with cLADs from 42% in wildtype to only 1% in LAP2α knockout myoblasts (Figure 4B, first and middle panel). Moreover, lamins moved away from (formerly) LAP2α-bound sites in LAP2α KO cells (Figure 4C).

Thus, there is a major rearrangement of lamin A/C chromatin binding in myoblasts in the absence of LAP2α, with lamins spreading towards active, cLAD-depleted euchromatic regions away from the genomic regions previously co-occupied with LAP2α. We concluded that in wildtype cells, LAP2α may maintain lamin A/C binding to specific genomic regions, preventing it from uncontrolled spreading to gene-rich euchromatic chromatin compartments.

**Figure 5.**
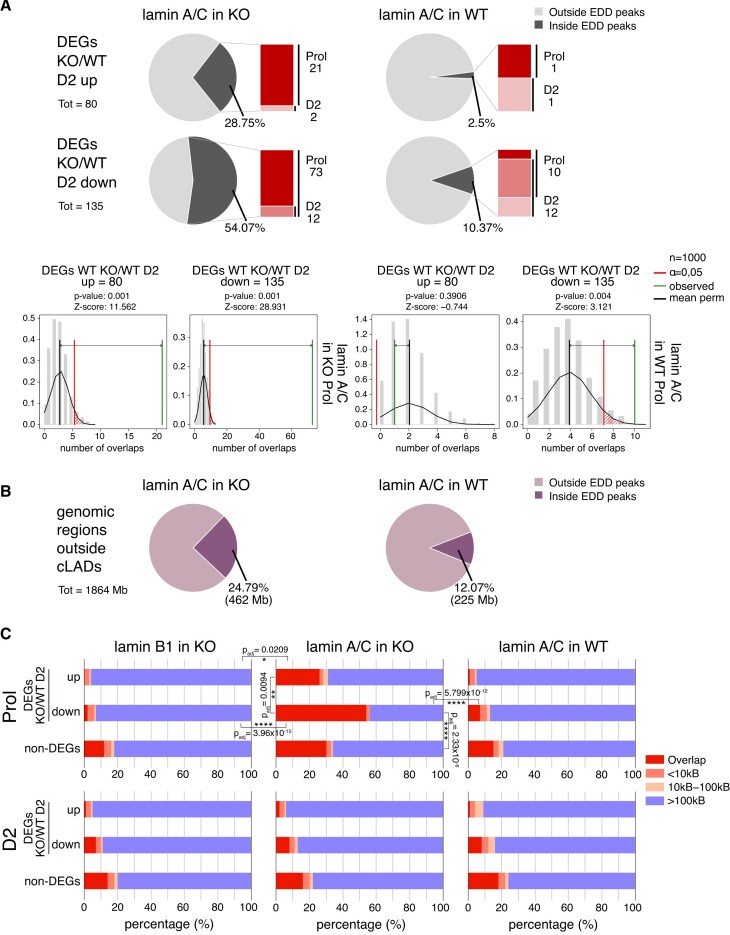
Lamin A/C relocalizes to chromatin regions overlapping with genes that are downregulated in LAP2α knockout cells. (**A**) Upper and middle panel: Pie charts showing the percentages of differentially expressed genes in LAP2α knockout versus wildtype myoblasts during differentiation (DEGs KO/WT D2; 215 genes) split into up- (80 genes; upper panel) and downregulated genes (135 genes; middle panel) that are found within or outside of lamin A/C EDD peaks in knockout (KO; left panel) and wildtype cells (WT; right panel). Bar charts on the right of each pie chart depict the number of DEGs found within EDD peaks in each differentiation stage (proliferating versus D2; dark versus light color, respectively; genes found in both stages are depicted in an intermediate color). Bottom panel: Lamin A/C EDD peaks for proliferating knockout (left panel) and wildtype myoblasts (right panel) were randomized 1000 times to yield genomic regions of the same size and the overlap with DEGs KO/WT D2 split into up- and downregulated genes was determined. The distribution of these randomized overlaps was plotted as histograms with the red line demarcating statistical significance at α=0.05 and the green line marking the observed overlap of DEGs with EDD peaks. Mean perm: Mean value for random permutation tests (black line). (**B**) Pie charts showing the percentages of genomic regions outside cLADs (1864 megabases) that are found within or outside of lamin A/C EDD peaks in knockout (KO; left panel) and wildtype cells (WT; right panel). Total lengths of overlapping regions are shown in brackets (Mb: megabases). (**C**) The genomic distances in basepairs of 215 DEGs KO/WT D2 (split into up- and downregulated genes) to the closest lamin A/C EDD peaks were determined in proliferating (Prol; upper panel) and differentiating (D2; lower panel) LAP2α knockout (middle panel) or wildtype myoblasts (right panel) and plotted in bar charts. The distances of non-differentially expressed genes (non-DEGs) to EDD peaks were also determined and plotted as a control. For LAP2α knockout myoblasts, the same analysis was performed for lamin B1 ChIP-seq EDD peaks (left panel). Statistically significant comparisons are indicated with * *P*< 0.05; **P< 0.01; ****P< 0.0001; *P*_adj_: *P*-value (KS test) adjusted for multiple testing. All other pairwise comparisons using the KS test were non-significant (*P*> 0.05).

To test whether lamin A/C also spreads towards genes that are up- or downregulated in LAP2α knockout myoblasts (DEGs KO/WT D2, 215 genes; see Figure 1B), we determined the fraction of these genes overlapping lamin A/C EDD peaks in wildtype and LAP2α knockout myoblasts (Figure 5A). Strikingly, whereas only a small minority of these genes overlapped with lamin A/C-bound chromatin in WT cells (2.5% and ∼10% of up- and downregulated genes, respectively; Figure 5A right panel), more than 50% of downregulated genes intersected with lamin A/C EDD peaks in LAP2α knockout cells, mainly in the proliferating stage (Figure 5A, left panel). This number was much higher than expected by chance as revealed by random permutation testing (Figure 5A, lower panel; Z-score = 30.221, *P* value = 0.001). The overlap of lamin A/C peaks with upregulated genes also increased from 2.5% in wildtype cells to ∼28% in LAP2α knockout cells (Figure 5A). However, when we intersected lamin A/C EDD peaks with all genomic regions outside cLADs, largely corresponding to the euchromatic A compartment (Figure 5B) (51,52), we observed a similar general overlap of euchromatin with lamin A/C in LAP2α knockout cells (Figure 5B; ∼25% in KO versus ∼12% in WT). Thus, whereas lamin A/C seems to generally spread across euchromatin in the absence of LAP2α, also including the upregulated genes in KO compared to wildtype cells, it specifically accumulates around downregulated genes in LAP2α knockout myoblasts, covering more than half of these 135 genes.

Moreover, when we determined the closest distance of each gene that was deregulated in LAP2α knockout myoblasts to the nearest lamin A/C EDD peak and compared this to the distance of peaks to all other genes (non-DEGs), lamin A/C clearly accumulated around the downregulated genes in LAP2α knockout cells (Figure 5C, middle panel; *P* value versus wildtype = 5.799 × 10^–12^; KS test), and this effect was significantly more pronounced compared to non-DEGs (Figure 5C; *P*-value = 2.33 × 10^–6^; KS test) and upregulated genes (*P*-value = 0.0094; KS test). In stark contrast, lamin A/C tended to avoid the deregulated genes in wildtype cells with only a small minority of genes overlapping lamin A/C peaks in proliferating and D2 cells (Figure 5C, right panel). Accumulation of lamin A/C in LAP2α knockout cells occurred on and around genes, without a particular enrichment on the gene bodies (Supplementary Figure S5B). Interestingly, the spreading of lamins to the deregulated genes occurred only transiently, as the gene overlap with lamin A/C EDD peaks was again low in LAP2α-depleted D2 myoblasts (Figure 5C, lower middle panel) and did not significantly differ from wildtype D2 cells. Spreading of lamin A/C across downregulated genes in LAP2α KO cells did not seem to relocate these genes to the nuclear periphery, as there was no or very low overlap with peripheral lamin B1 bound genomic regions in both proliferating and D2 KO myoblasts (Figure 5C, left panel).

Overall, we conclude that lamin A/C seems to avoid regions containing deregulated genes in proliferating wildtype myoblasts. Upon LAP2α depletion, lamin A/C transiently spreads to euchromatic regions, particularly covering areas containing downregulated genes. Consequently, these genes are not properly expressed during early stages of differentiation, suggesting that the uncontrolled spreading of lamin A/C to these genes in LAP2α-depleted cells may interfere with proper gene regulation.

### Lamin A/C accumulates at H3K27ac-enriched genomic regions in LAP2α knockout cells concomitant with a depletion of H3K27ac histone marks and downregulation of genes

We reasoned that epigenetic alterations may be one possible mechanism by which mislocalized lamin A/C might interfere with proper gene regulation. This is consistent with previous studies showing that LAP2α loss coincided with epigenetic changes in fibroblasts (11) and that the effects of several lamin A mutations in different cell systems were linked to epigenetic alterations (22,53,54). To test if and how relocalization of lamin A/C on chromatin in the absence of LAP2α might affect epigenetic modifications and proper gene regulation, we identified publicly available histone modification ChIP-seq data sets (from the CISTROME database) (47) that were most similar to lamin A/C EDD peaks in knockout and wildtype myoblasts (Supplementary Figure S6). Remarkably, whereas lamin A/C EDD peaks in wildtype cells were mostly enriched in repressive histone marks, including H3K9me3, H3K27me3 and H3K9me2, the similarity search in knockout cells retrieved an unusually high number of active marks, including the promoter and enhancer marks H3K4me3, H3K27ac and H3K4me1 (Supplementary Figure S6, middle panel). Notably, the same analysis performed with LAP2α EDD peaks revealed very low similarities with most histone modifications, although the promoter mark H3K4me3 came up as the top hit (Supplementary Figure S6, lower right panel).

**Figure 6.**
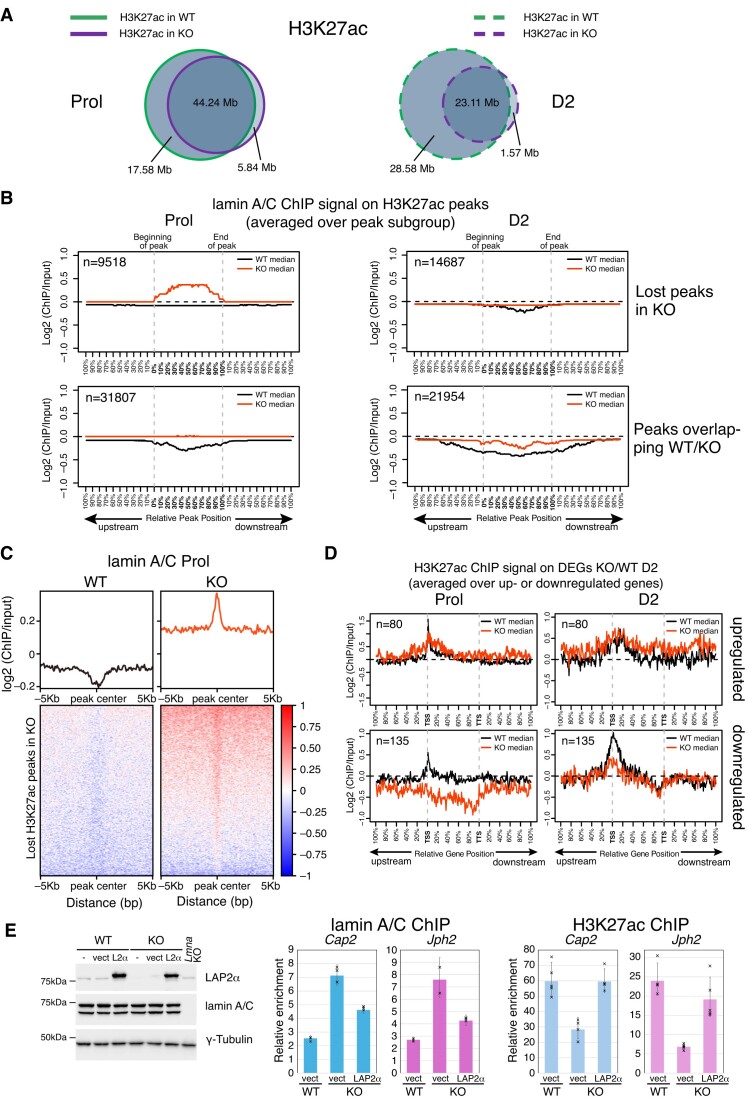
Accumulation of lamin A/C correlates with loss of active H3K27ac histone marks in LAP2α knockout cells. (**A**) ChIP-seq analysis was performed in wildtype (WT) and LAP2α knockout (KO) myoblasts using an H3K27ac-specific antibody. Venn diagrams depict the overlap of H3K27ac ChIP peaks in wildtype (green) and LAP2α knockout (purple) proliferating (Prol; left panel, solid lines) and differentiating cells (D2; right panel, dashed lines). The total genomic lengths of overlapping and non-overlapping regions between peak sets were identified using the intersect function of the BEDTools suite and are shown in megabases (Mb). (**B**) Median log2 ratio tracks (ChIP over input) of lamin A/C in proliferating (Prol) and differentiating (D2) wildtype (WT; black) and LAP2α knockout myoblasts (KO; red) were plotted on H3K27ac ChIP-seq peaks subdivided into peaks that are lost upon depletion of LAP2α (lost peaks in KO; upper panel) and those that are maintained (peaks overlapping WT/KO; lower panel). Peak lengths were normalized by scaling (full length of peak = 100%). One peak-length upstream of the beginning of each peak and downstream of the end of each peak was also plotted. (**C**) Heat maps displaying log2 ratio signal (ChIP over input) for lamin A/C in proliferating (Prol) wildtype (left panel) and LAP2α knockout myoblasts (right panel) on H3K27ac ChIP-seq peaks that are lost in KO cells. Graphs on top of heatmaps show mean log2 ratio tracks. (**D**) Median H3K27ac ChIP log2 ratio tracks (ChIP over input) in proliferating (left panel) and differentiating (D2; right panel) wildtype (black) and LAP2α knockout (red) myoblasts on deregulated genes in LAP2α knockout (DEGs KO/WT D2; 215 genes) split into up- and downregulated genes are shown. Gene lengths were normalized by scaling (full length of gene = 100%). One gene-length upstream of the TSS (transcription start site) and downstream of the TTS (transcription termination site) was also plotted. (**E**) Left panel: WT and LAP2α KO myoblasts were either left untreated (-), transduced with an empty lentiviral vector (vect) or with the same vector encoding FLAG-tagged wildtype LAP2α (L2α), followed by Western blot analysis using the indicated antibodies. Lamin A/C knockout (*Lmna* KO) myoblasts were included as a control. Right and middle panel: ChIP-qPCR analysis was performed in vector-transduced WT and LAP2α KO cells, as well as LAP2α KO cells expressing wildtype LAP2α, using antibodies to lamin A/C (middle) and H3K27ac (right). Precipitated chromatin was analyzed using primers specific to the regulatory region (±1kB up- and downstream of TSS) of two genes (*Cap2, Jph2*) that are downregulated in LAP2α KO cells. Data are displayed as average fold enrichment of specific ChIP/IgG control ± standard deviation of 3 (lamin A/C) and 5 (H3K27ac) technical replicates of a representative experiment. Single data points are depicted for each column.

To further test the effect of LAP2α loss-mediated lamin A/C relocalization on chromatin on the distribution of the active enhancer and promoter mark, H3K27ac, we performed ChIP-seq using H3K27ac antibodies in wildtype and LAP2α-depleted myoblasts. Interestingly, LAP2α knockout cells lost between 28% (in proliferating cells) and 55% (in differentiating myoblasts) of H3K27ac peaks compared to wildtype cells, whereas only very few peaks were gained in the absence of LAP2α (Figure 6A). To investigate a potential direct involvement of lamin A/C relocalization on chromatin in the loss of H3K27ac marks upon LAP2α depletion, we plotted the average lamin A/C ChIP-seq log2 ratio in wildtype and LAP2α knockout cells on both, H3K27ac peaks that are lost upon depletion of LAP2α and those that are maintained (Figure 6B). Strikingly, in the absence of LAP2α we observed a significant accumulation of lamin A/C on the lost H3K27ac peaks, i.e. on positions that are occupied by H3K27ac in wildtype but not in knockout myoblasts (Figure 6B, upper left panel). This accumulation of lamin A/C was particularly pronounced in the proliferating stage, where lamin A/C spreading on euchromatin was most evident (see Figure 6B and Figure 4A and B). H3K27ac peaks that were maintained in the absence of LAP2α (overlapping peaks WT/KO) had no clear accumulation of A-type lamins in knockout cells; however, the overall depletion of lamin A/C on these positions visible in wildtype cells was less pronounced in LAP2α-depleted cells (Figure 6B, lower panel). Heat maps of lamin A/C ChIP-seq log2 ratios on the lost H3K27ac peaks in LAP2α knockout cells confirmed the strong accumulation of A-type lamins around the peak center in LAP2α knockout cells, whereas the lamin signal was depleted on these peaks in wildtype cells (Figure 6C).

To address the potential relevance of these lost H3K27ac marks for the deregulation of genes in LAP2α knockout cells (DEGs KO/WT D2), we plotted the average H3K27ac ChIP-seq log2 ratio on up- and downregulated genes in LAP2α knockout versus wildtype myoblasts (Figure 6D). Interestingly, the H3K27ac signal was significantly reduced on downregulated genes at the TSS and on the gene body in both, proliferating and differentiating LAP2α knockout cells when compared to wildtype (Figure 6D, lower panel, and Supplementary Figure S7A), whereas the H3K27ac signal remained largely unaltered on upregulated genes (Figure 6D, upper panel). Together these data indicate that the loss of H3K27ac marks contributed to the downregulation of genes in LAP2α knockout cells.

Altogether, lamin A/C relocates to regions enriched in active H3K27ac marks in the absence of LAP2α, concomitant with a general reduction of H3K27ac marks on and around downregulated genes. It is thus tempting to speculate that accumulation of lamin A/C on H3K27ac-enriched sites may contribute to the loss of this histone modification, probably by recruiting histone deacetylases. In support of this notion, regulatory regions around the transcription start site of two downregulated genes (*Cap2 and Jph2*) displayed increased lamin A/C accumulation in LAP2α knockout cells, together with an increased binding of the histone deacetylase HDAC1 compared to wildtype cells, as demonstrated by ChIp-qPCR (Supplementary Figure S7B).

Notably, re-expression of LAP2α in LAP2α knockout myoblasts reduced lamin A/C accumulation on these regulatory regions of the downregulated genes, with a concomitant increase of the H3K27ac signal back to wildtype levels (Figure 6E). Altogether these data demonstrate that both, euchromatic spreading of lamin A/C and loss of H3K27ac on downregulated genes are direct and specific consequences of the depletion of LAP2α.

### Accumulation of lamin A/C does not affect H3K4me3 histone marks in LAP2α knockout cells

**Figure 7.**
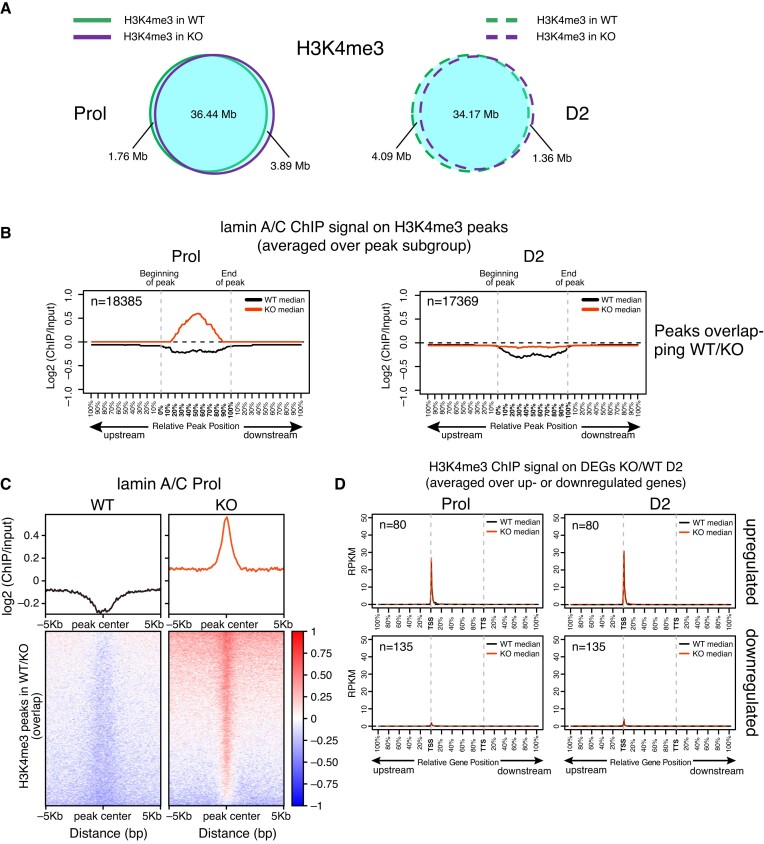
H3K4me3 histone marks are not affected by accumulation of lamin A/C in LAP2α knockout cells. (**A**) ChIP-seq analysis was performed in wildtype (WT) and LAP2α knockout (KO) myoblasts using an H3K4me3-specific antibody. Venn diagrams depict the overlap of H3K4me3 ChIP peaks in wildtype (green) and LAP2α knockout (purple) proliferating (Prol; left panel, solid lines) and differentiating cells (D2; right panel, dashed lines). The total genomic lengths of overlapping and non-overlapping regions between peak sets were identified using the intersect function of the BEDTools suite and are shown in megabases (Mb). (**B**) Median log2 ratio tracks (ChIP over input) of lamin A/C in proliferating (Prol) and differentiating (D2) wildtype (WT; black) and LAP2α knockout myoblasts (KO; red) were plotted on H3K4me3 ChIP-seq peaks overlapping in WT and LAP2α KO cells. Peak lengths were normalized by scaling (full length of peak = 100%). One peak-length upstream of the beginning of each peak and downstream of the end of each peak was also plotted. (**C**) Heat maps displaying log2 ratio signal (ChIP over input) for lamin A/C in proliferating (Prol) wildtype (left panel) and LAP2α knockout myoblasts (right panel) on H3K4me3 ChIP-seq peaks overlapping in WT and LAP2α KO cells. Graphs on top of heatmaps show mean log2 ratio tracks. (**D**) Median H3K4me3 ChIP log2 ratio tracks (ChIP over input) in proliferating (left panel) and differentiating (D2; right panel) wildtype (black) and LAP2α knockout (red) myoblasts on deregulated genes in LAP2α knockout (DEGs KO/WT D2; 215 genes) split into up- and downregulated genes are shown. Gene lengths were normalized by scaling (full length of gene = 100%). One gene-length upstream of the TSS (transcription start site) and downstream of the TTS (transcription termination site) was also plotted.

We also performed ChIP-seq for the active promoter mark H3K4me3 in wildtype and LAP2α knockout myoblasts, another histone modification retrieved in the similarity search for lamin A/C peaks (Supplementary Figure S6 and Figure 7A). Interestingly, we did not observe major changes in the number or localization of H3K4me3 peaks in the presence versus absence of LAP2α (Figure 7A), although a similar enrichment of lamin A/C as observed for H3K27ac sites was found on the H3K4me3 peaks (Figure 7B and C). Similarly, the H3K4me3 signal was unchanged on the deregulated genes in LAP2 knockout versus wildtype cells (Figure 7D and Supplementary Figure S7C).

Altogether, we observed a strong accumulation of lamin A/C on genomic regions enriched for active H3K4me3 and H3K27ac histone marks in LAP2α knockout versus wildtype cells. However, while H3K4me3 marks were maintained in lamin A/C-bound regions, the accumulation of lamin A/C on H3K27ac-enriched regions led to loss of this mark on and around genes and was associated with downregulation of these genes.

In summary, our genome-wide analyses provide novel insights into the role of LAP2α in muscle differentiation. In wildtype cells, LAP2α may prevent the spreading of lamin A/C towards myogenic genes by so far unknown mechanisms. Furthermore, relocalization of LAP2α towards gene-rich genomic regions containing a subset of myogenic genes in early stages of muscle differentiation may facilitate efficient gene regulation. Upon loss of LAP2α, lamin A/C spreads towards active chromatin accumulating at H3K27ac and H3K4me3-enriched regions close to the deregulated genes in LAP2α knockout myoblasts. The lamin A/C enrichment is accompanied by H3K27ac depletion (leaving H3K4me3 unaffected), which likely influences the expression of nearby genes. Ultimately, these epigenetic and gene expression changes may underlie the delayed differentiation observed in LAP2α knockout muscle cells and tissues.

## Discussion

Here, we show that chromatin association of LAP2α regulates proper myogenic differentiation. LAP2α prevents the spreading of nucleoplasmic lamin A/C to regulatory elements of myogenic genes, allowing their timely and unperturbed expression. Additionally, the relocation of LAP2α to genomic regions containing myogenic genes may facilitate efficient gene regulation during early stages of muscle differentiation.

LAP2α was previously proposed to function during the transition of cells from one cell state to another, for example when exiting the cell cycle or initiating differentiation. Overexpression of LAP2α delayed cell cycle reentry from a non-proliferating state in fibroblasts and, in proliferating preadipocytes it caused premature adipogenic differentiation *in vitro* (55). *In vivo*, depletion of LAP2α in mice led to increased proliferation of muscle tissue progenitor cells and delayed myogenic differentiation (13,14). Based on reports suggesting a direct interaction of LAP2α and the tumor suppressor Retinoblastoma protein (pRb) (56), it was proposed that LAP2α might affect cell cycle exit and differentiation by regulating pRb function in repressing pRb target genes. Interestingly, LAP2α was also found to positively affect the activity of other transcriptional regulators, such as GLI1 and MRTF-A (57,58). Of note, both pRb and MTRF-A have essential roles during muscle differentiation (59,60). Our new finding that LAP2α relocates towards myogenic genes in early myogenic differentiation is consistent with a more general function of LAP2α in modulating transcriptional regulators. LAP2α may fulfill this function by either directly assisting the association of transcriptional regulators with their target genes (55) or by providing a structural scaffold for the formation of transcriptional complexes (56–58), but mechanistic details remain mostly elusive.

Additionally, as our results show, LAP2α may facilitate proper gene expression by regulating the localization and functions of nucleoplasmic lamin A/C. LAP2α and lamin A/C form dynamic complexes in the nucleoplasm and associate with largely overlapping regions on euchromatin in fibroblasts (9,11). In the absence of LAP2α, lamin A/C forms larger, biochemically stable and less mobile structures in the nuclear interior (9), which seems to also affect association of lamin A/C with chromatin as shown in our genome-wide lamin A/C ChIP-seq analyses. We find spreading of A-type lamins towards euchromatic genomic regions in LAP2α knockout myoblasts compared to wildtype. Strikingly, these genomic regions, newly bound by lamin A/C in LAP2α knockout myoblasts, cover over 50% of the genes downregulated upon LAP2α depletion, suggesting that the aberrant spreading of lamin A/C to these genes might interfere with their proper expression. While we could not observe direct binding of lamin A/C to deregulated genes, lamins particularly accumulated at chromatin elements carrying the active histone marks H3K4me3 and H3K27ac in close vicinity to these genes, which are typically found on active promoters and enhancers (61).

Intriguingly, we observed a strong reduction of the active H3K27ac histone mark on the newly lamin A/C-bound chromatin sites in LAP2α knockout myoblasts, whereas the H3K4me3 marks remained unaffected. These results are in line with earlier findings that changes in lamin A/C chromatin binding upon loss of LAP2α globally affect the epigenetic landscape in fibroblasts (11) and that expression of disease-linked mutants of lamin A in various cell types correlates with changes in histone modifications (21,22,53,54). It is thus tempting to speculate that the increased binding of lamin A/C to active, H3K27ac-enriched chromatin regions in LAP2α knockout myoblasts may interfere with the setting of this mark. Notably, we observed increased binding of the histone deacetylase HDAC1 to sites, where lamin A/C accumulates in LAP2α knockout cells. As lamin A/C was found to bind to HDACs in muscle cells (62,63), it is possible that lamin A/C recruits HDAC1 to H3K27ac-enriched sites leading to H3K27ac loss. Alternatively, lamin A/C could affect the binding and/or activity of the histone acetyl transferase CBP/p300, which catalyzes the H3K27ac modification at active enhancers (64). However, we cannot exclude the possibility that the reduction in H3K27ac is not a direct consequence of lamin A/C accumulation but is due to other yet unknown reasons.

Intriguingly, loss of the H3K27ac signal was most pronounced on genes that are downregulated in LAP2α knockout cells compared to wildtype, suggesting that the H3K27ac histone mark is involved in the correct regulation of these myogenic genes. As H3K27ac is essential for enhancer activation and associated gene expression (65,66), the observed reduction in H3K27ac histone marks upon lamin A/C enrichment in LAP2α knockout myoblasts may interfere with efficient activation of these myogenic genes, ultimately causing an impaired myogenic differentiation. In wildtype myoblasts active, H3K27ac-enriched chromatin regions were largely devoid of lamin A/C, fitting to the notion that lamins generally reside in a repressive chromatin environment (67). A few studies reported association of lamin A/C also with promoters and enhancers, but this was usually correlated with transcriptional repression (19,21,22). The only exception so far is a report showing that specifically lamin A/C phosphorylated on S22 is found on active enhancers carrying the H3K27ac mark (20). In our ChIP-seq analyses we used a pan-lamin A/C antibody recognizing both, phosphorylated and non-phosphorylated forms of lamin A/C, but we did not find an enrichment of lamin A/C on active enhancers in wildtype cells. Also, our lamin A/C ChIP analyses in myoblasts using an anti-pS22 lamin A/C antibody revealed mostly binding to repressive chromatin regions (data not shown). However, we cannot fully exclude that lamin A/C accumulating at regulatory elements in LAP2α knockout cells may represent pS22 lamin A/C.

Altogether, altered gene regulation and impaired myogenic differentiation in LAP2α knockout myoblasts can be explained by at least two different, non-mutually exclusive mechanisms: Firstly, LAP2α may have an active role in transcriptional regulation of myogenic genes by relocating towards regions around these genes in early stages of differentiation, allowing efficient binding of transcriptional regulators to promoters and enhancers linked to these genes. The specific mechanisms of such a function remain elusive, but it is tempting to speculate that they may be linked to the formation of phase-separated compartments of LAP2α on active chromatin, based on the largely unstructured regions of the LAP2α polypeptide and its high probability score to undergo phase separation (our unpublished data and (68)). Secondly, LAP2α may prevent uncontrolled spreading of lamin A/C across euchromatin, which likely interferes with proper gene regulation. This mechanism is supported by our finding that newly lamin A/C-bound genomic regions in LAP2α knockout cells covered more than 50% of the genes downregulated in the absence of LAP2α. In addition, satellite cell proliferation and myogenesis were improved in LAP2α-lamin A/C double knockout mice compared to single knockouts (69).

What could be the mechanisms by which LAP2α restricts lamin A/C binding to active chromatin? One can envisage different possibilities: Since LAP2α affects lamin A/C assembly *in vitro* and *in vivo* (9), it is possible that larger, higher-order lamin A/C complexes in the nucleoplasm in LAP2α knockout cells may associate more stably with chromatin genome-wide. Alternatively, LAP2α could restrict lamin A/C chromatin binding more directly, for example by competing with lamin A/C for chromatin binding or by forming spatially confined condensates on chromatin that may exclude lamin A/C from specific genomic regions, while concentrating it in others. In support of such a mechanism, we recently found that different complexes of LAP2α and lamin A/C compete for efficient chromatin binding without direct interaction of these proteins (27).

If lamin A/C has to be excluded from active gene-rich genomic regions to allow proper gene regulation, what is the specific function of euchromatin-bound nucleoplasmic lamin A/C complexes in wildtype conditions? Lamin A/C seems to bind to mostly gene-depleted euchromatic regions throughout myoblast differentiation, making it less likely to have a direct role in myogenic gene regulation. It is possible that lamin A/C forms a nucleoplasmic structural scaffold supporting three-dimensional organization of chromatin throughout the nucleus. Notably, depletion of lamin A/C in fibroblasts led to a significant increase in chromatin diffusion in the nuclear interior, possibly by disrupting lamin-mediated chromosomal inter-chain interactions throughout the nucleus (70). The spreading of lamins to gene-rich euchromatic regions in the absence of LAP2α would then represent an unwanted gain-of-function of lamin A/C in altering gene expression, showing that a well-balanced interplay of LAP2α and lamin A/C, restricting lamin A/C binding to gene-rich regions along with allowing formation of chromatin scaffolds in other regions is essential for efficient gene regulation.

On the whole, our study provides important novel insights into the relationship of LAP2α and nucleoplasmic lamin A/C in gene regulation and myogenic differentiation. As absence of LAP2α affects differentiation of progenitor cells in several tissues, including skin, colon and hematopoietic cells (13), similar mechanisms of LAP2α-mediated gene regulation might apply to other tissues as well. These findings will also help in understanding disease mechanisms in laminopathies, a group of human diseases caused by mutation in the LMNA gene (24). Notably, particularly muscle tissue is heavily affected in laminopathy patients. Most studies so far have investigated the role of the peripheral nuclear lamina in the development of laminopathic diseases, but little is known about the contribution of the nucleoplasmic lamin A/C pool. Since our study shows involvement of nucleoplasmic lamin A/C and LAP2α in early myoblast differentiation, it is tempting to speculate that perturbations of these functions contribute to the altered proliferation and differentiation of muscle satellite cells in patients (14,15,17,71), which ultimately affects the regenerative potential of muscle tissue.

## Supplementary Material

gkae752_Supplemental_Files

## Data Availability

The data underlying this article are available in the Gene Expression Omnibus (GEO) at https://www.ncbi.nlm.nih.gov/geo/, and can be accessed with the GEO accession number GSE247774.

## Notes

Present address: Celine Prakash, St. Anna Children's Cancer Research Institute (CCRI), Vienna 1090, Austria.
