# Supplementary material for: LAP2alpha facilitates myogenic gene expression by preventing nucleoplasmic lamin A/C from spreading to active chromatin regions
Source: Nucleic Acids Res. 2024 Sep 4;52(19):11500–18. doi: 10.1093/nar/gkae752 (PMC11514464; doi:10.1093/nar/gkae752)
Supplement: gkae752_Supplemental_Files [file gkae752_supplemental_files.zip › NAR-supplements-Ferraioli et al_revised_figures embedded.pdf]

SUPPLEMENTARY DATA

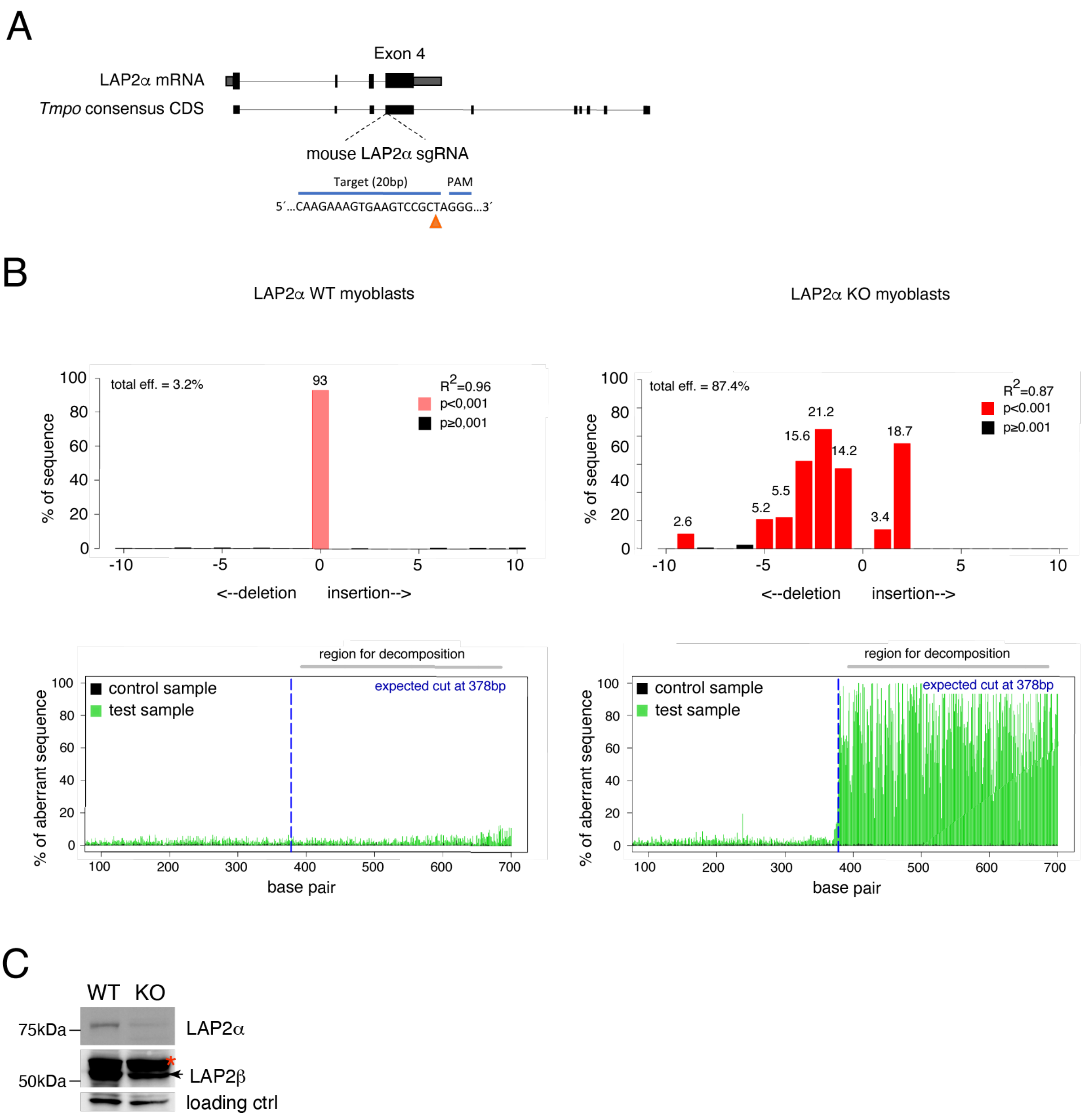

Figure S1. **Generation of LAP2α knockout myoblasts using CRISPR-Cas9. (A)** Top: Schematic view of mouse LAP2α mRNA (top) and *Lap2/Tmpo* gene locus (bottom) with exons 1-4 (bars) and adjacent introns (lines). CDS: coding sequence. Position of the target sequence of LAP2α-specific sgRNA at the beginning of exon 4 is shown. Protospacer-adjacent motif (PAM) sequence is indicated. Red arrowhead: expected Cas9 cut site. **(B)** A region spanning Cas9 cut site was amplified from genomic DNA of LAP2α knockout (KO) and wildtype (WT) control cells by PCR. PCR products were sequenced and analyzed using TIDE software (26).

Upper panel: Detected indels and their frequency in % (numbers atop red bars). Lower panel: Alignment of sequences from control sample (untransfected wildtype myoblasts) with wildtype sample (vector-transfected) and LAP2α knockout sample (sgRNA-transfected). Frequency of aberrant sequences, defined as sequences that differ from control, is displayed on the Y axis and increases drastically in knockout cells after indel-induced frame shifts at the expected cut site (dashed blue line). The region used for decomposition of individual sequences and indel prediction is marked in grey. **(C)** WT and LAP2α KO myoblasts were processed for Western blot analysis using an antibody against the N-terminal domain common to all LAP2 isoforms. Depicted are the LAP2α and β isoforms as indicated. Red asterisk denotes an unspecific band atop of LAP2β. A weak unspecific background band was used as loading control.

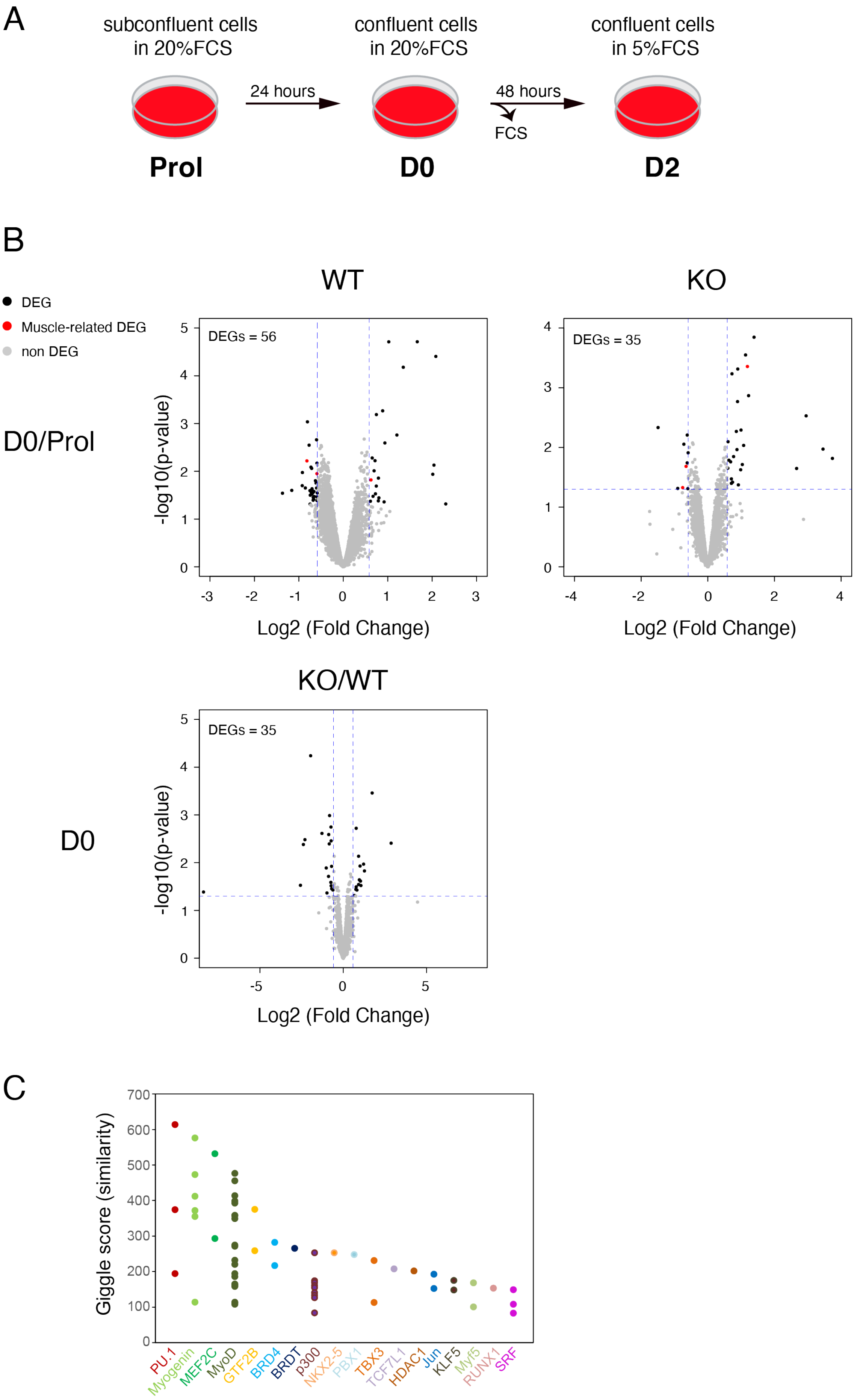
A
subconfluent cells in 20%FCS
24 hours
confluent cells in 20%FCS
48 hours
FCS
confluent cells in 5%FCS
Prol
D0
D2
B
DEG
Muscle-related DEG
non DEG
D0/Prol
WT
KO
DEGs = 56
DEGs = 35
-log10(p-value)
Log2 (Fold Change)
D0
KO/WT
DEGs = 35
-log10(p-value)
Log2 (Fold Change)
C
Giggle score (similarity)
PU.1
Myogenin
MEF2C
MyoD
GTF2B
BRD4
BRDT
p300
NKX2-5
PBX1
TBX3
TCF7L1
HDAC1
Jun
KLF5
Myf5
RUNX1
SRF

Figure S2. **Characterization of myoblast differentiation stages in wildtype and LAP2α knockout cells. (A)** Schematic overview of myoblast differentiation stages. Myoblasts were seeded on culture dishes at subconfluent conditions for proliferating (Prol) samples. After 24hrs cells were reaching confluency corresponding to day 0 of differentiation (D0), upon which serum was withdrawn (reduction from 20% to 5% serum) for 48 hours to reach day 2 of differentiation (D2). FCS: fetal calf serum. **(B)** Wildtype (WT) and LAP2α knockout (KO) myoblasts were differentiated according to the scheme in (A), followed by RNA isolation and RNA-sequencing. Volcano plots display differentially expressed genes (DEGs) in wildtype D0 versus proliferating (Prol) cells (upper left panel) and knockout D0 versus proliferating cells (upper right panel). Additionally, DEGs in LAP2α knockout versus wildtype D0 cells were analyzed. Significantly differentially expressed genes are depicted in black. Genes related to muscle differentiation are depicted in red. Non-significantly changed genes are depicted in grey. **(C)** Giggle software was used to search the Cistrome database and return transcription factor ChIP-seq peak data sets that are most enriched within the regulatory region (1kB upstream of transcription start site) of 63 unique DEGs KO/WT D2 found in the top 10 GO terms as shown in Figure 1D. Graph displays Giggle scores as measure of similarity/enrichment on the Y axis for the top-ranked transcriptional regulators. Number of dots corresponds to number of datasets found for the indicated factor.

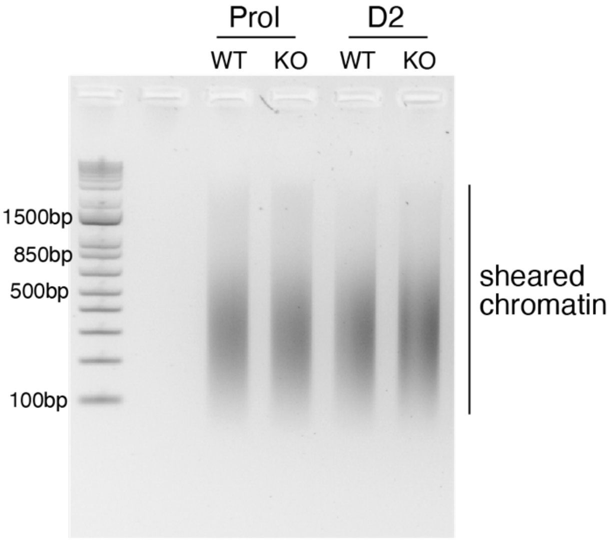

Figure S3. **Mild sonication of formaldehyde-crosslinked chromatin yields fragment sizes between 100bp and 800bp.** Formaldehyde-crosslinked chromatin from proliferating (Prol) and differentiating (differentiation D2) wildtype (WT) and LAP2α knockout (KO) myoblasts was sonicated using the Bioruptor PICO sonication device (see Materials and Methods for details). Sheared chromatin was de-crosslinked, purified and fragment size distribution was analyzed on a 2% agarose gel. Size of DNA marker bands in base pairs (bp) is indicated on the left.

A

ChIP sample	# of peaks	Total peak length (Mb)	Mean peak length (Mb)	Genome coverage (%)	Pearson's R (replicates)
LAP2α Prol	115	426.9	3.71	15.7	0.89
LAP2α D2	131	313.04	2.39	11.5	0.88
WT LAC 3A6 Prol	337	415.7	1.23	15.3	0.88
WT LAC 3A6 D2	459	542.7	1.18	19.9	0.88
WT LAC E1 Prol	424	481.4	1.14	17.7	n.d.
WT LAC E1 D2	512	592.17	1.16	21.7	n.d.
KO LAC 3A6 Prol	248	470.27	1.90	17.3	0.87
KO LAC 3A6 D2	346	442.71	1.28	16.2	0.88

B

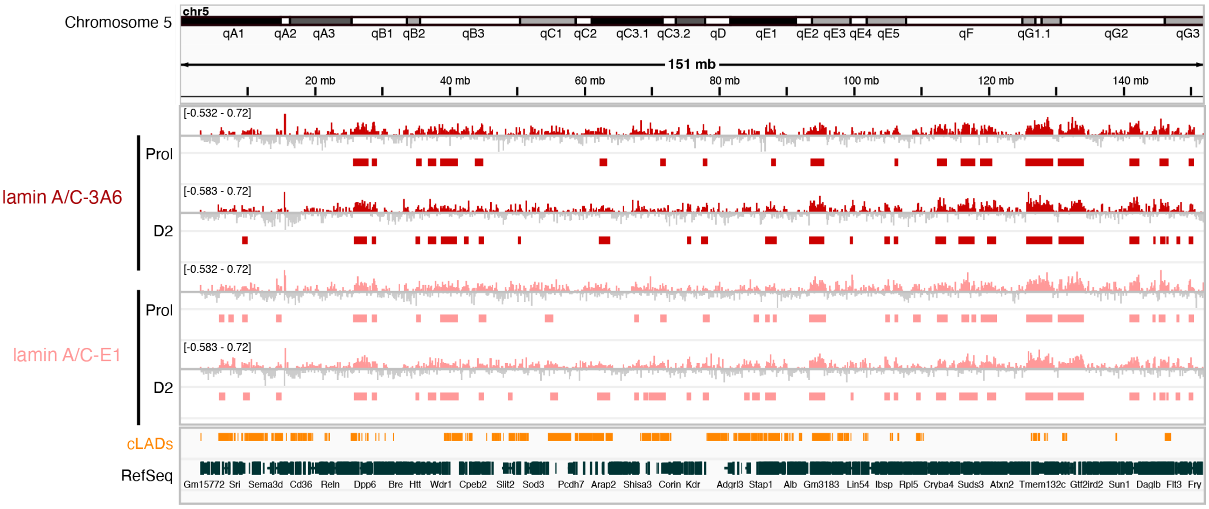

C

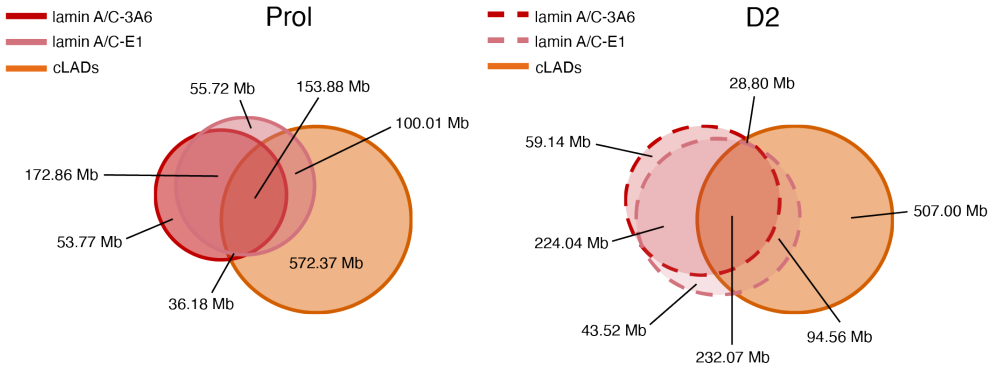

Figure S4. **Lamin A/C ChIP-seq peaks using antibodies to the C-terminus or N-terminus of lamin A/C largely overlap. (A)** Table depicting a summary of main parameters for LAP2α and lamin A/C ChIP-seq and Pearson correlation coefficient of ChIP replicates. **(B)** ChIP-seq analysis was performed in proliferating (Prol) and differentiating wildtype immortalized myoblasts (D2: day 2 of differentiation) using an antibody to the C-terminus (3A6; dark red) or to the N-terminus of lamin A/C (E1; light red) as indicated. IGV browser was used to display

log2 ratio signal tracks (ChIP over input) of mouse chromosome 5. Positive log 2 ratio values are depicted in color, negative values in grey. Peaks called by the Enriched Domain Detector software (EDD) are depicted for each ChIP track. The scale of each log2 ratio track is indicated on the left. cLADs: constant lamina-associated domains. RefSeq: Gene annotations from the NCBI reference sequence database. **(C)** Venn diagrams depicting the overlap of lamin A/C ChIP EDD peaks using the antibody to the C-terminus (3A6; dark red circle) or to the N-terminus of lamin A/C (E1; light red circle) in proliferating cells (Prol; left panel, solid lines) and differentiating cells (D2; right panel, dashed lines). Additionally, the overlap with cLAD regions is displayed (orange circle). The total genomic lengths of overlapping and non-overlapping regions between peak sets were identified using the intersect function of the BEDTools suite and are shown in megabases (Mb).

A

EDD peaks	DEGs D2/Prol (WT) Outside cLADs		Inside cLADs	
	Overlapping	Bound	Overlapping	Bound
LAP2α Prol	221	33	11	1
LAP2α D2	806	32	1	0
lamin A/C Prol	154	0	2	0
lamin A/C D2	163	0	4	0

B

EDD peaks	WT DEGs KO/WT (D2) Outside cLADs		Inside cLADs		KO DEGs KO/WT (D2) Outside cLADs		Inside cLADs	
	Overlapping	Bound	Overlapping	Bound	Overlapping	Bound	Overlapping	Bound
LAP2α Prol	9	1	1	0				
LAP2α D2	37	1	0	0				
lamin A/C Prol	11	0	0	0	94	0	0	0
lamin A/C D2	13	0	0	0	13	0	0	0

Figure S5. **LAP2α and lamin A/C do not directly bind to genes differentially expressed during myoblast differentiation or deregulated genes in LAP2α knockout cells.** Tables showing **(A)** differentially expressed genes in wildtype differentiating (D2) versus proliferating myoblasts (DEGs D2/Prol WT; total of 4257 genes) or **(B)** deregulated genes in LAP2α knockout cells (DEGs KO/WT D2; 215 genes), overlapping with LAP2α or lamin A/C EDD peaks in proliferating and differentiating myoblasts inside or outside of cLADs as indicated. The number of genes directly bound by LAP2α or lamin A/C is reported (see "Materials and methods" for definition of directly bound genes).

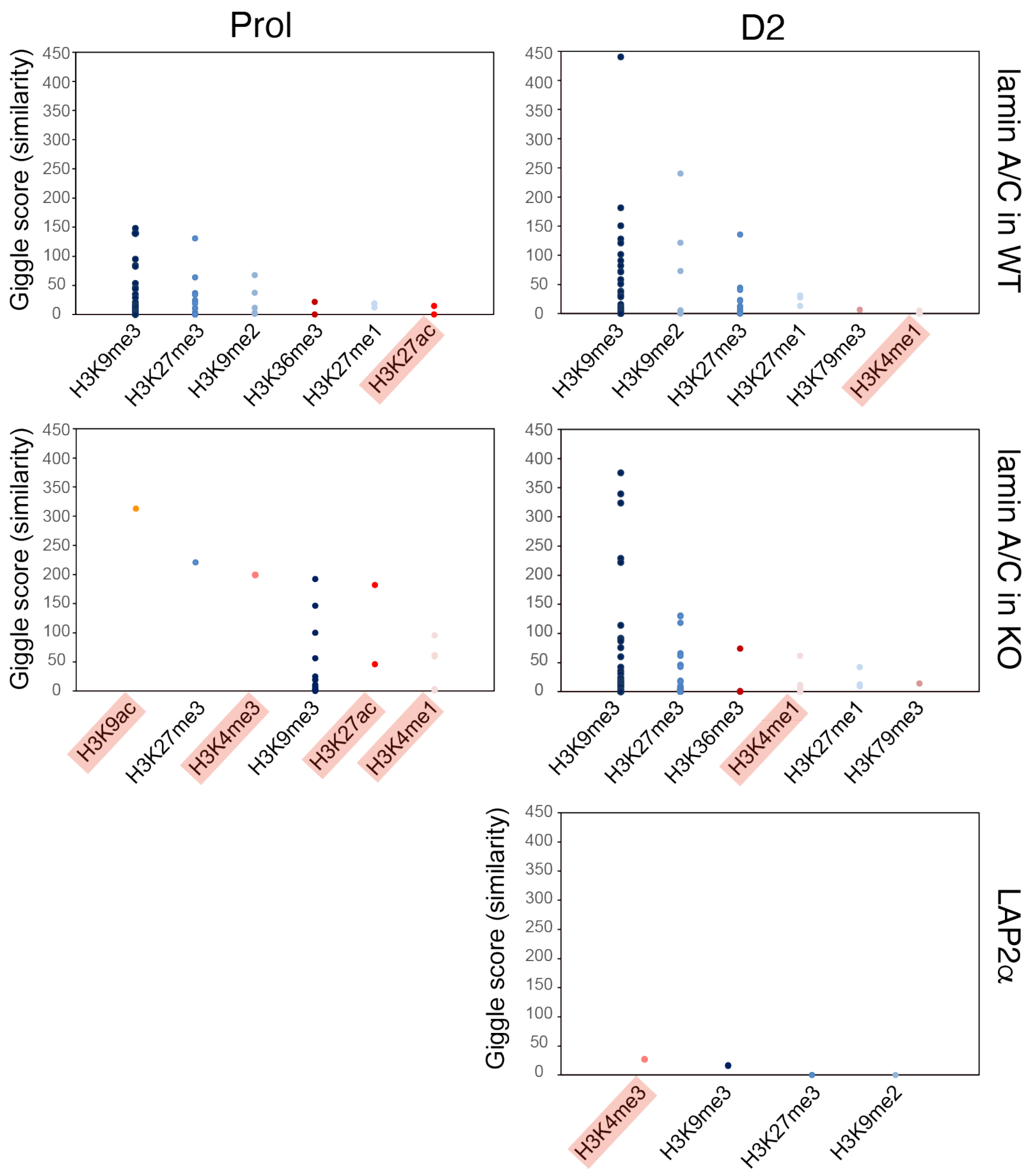

Figure S6. **In LAP2α knockout cells lamin A/C relocates to chromatin regions enriched in active histone marks.** Giggle software (42) was used to search the Cistrome database (46) and return histone modification ChIP-seq peak data sets that are most similar to lamin A/C ChIP-seq EDD peaks in wildtype (WT; upper panel) and LAP2α knockout (KO; middle panel) proliferating (Prol; left) and differentiating myoblasts (D2; right), or LAP2α ChIP-seq EDD peaks in D2 wildtype cells (lower panel). Graphs display Giggle score as measure of similarity on the Y axis for the top-ranked 6 histone modifications. Number of dots corresponds to number of datasets found for the indicated histone modification. Active histone marks are shaded in red.

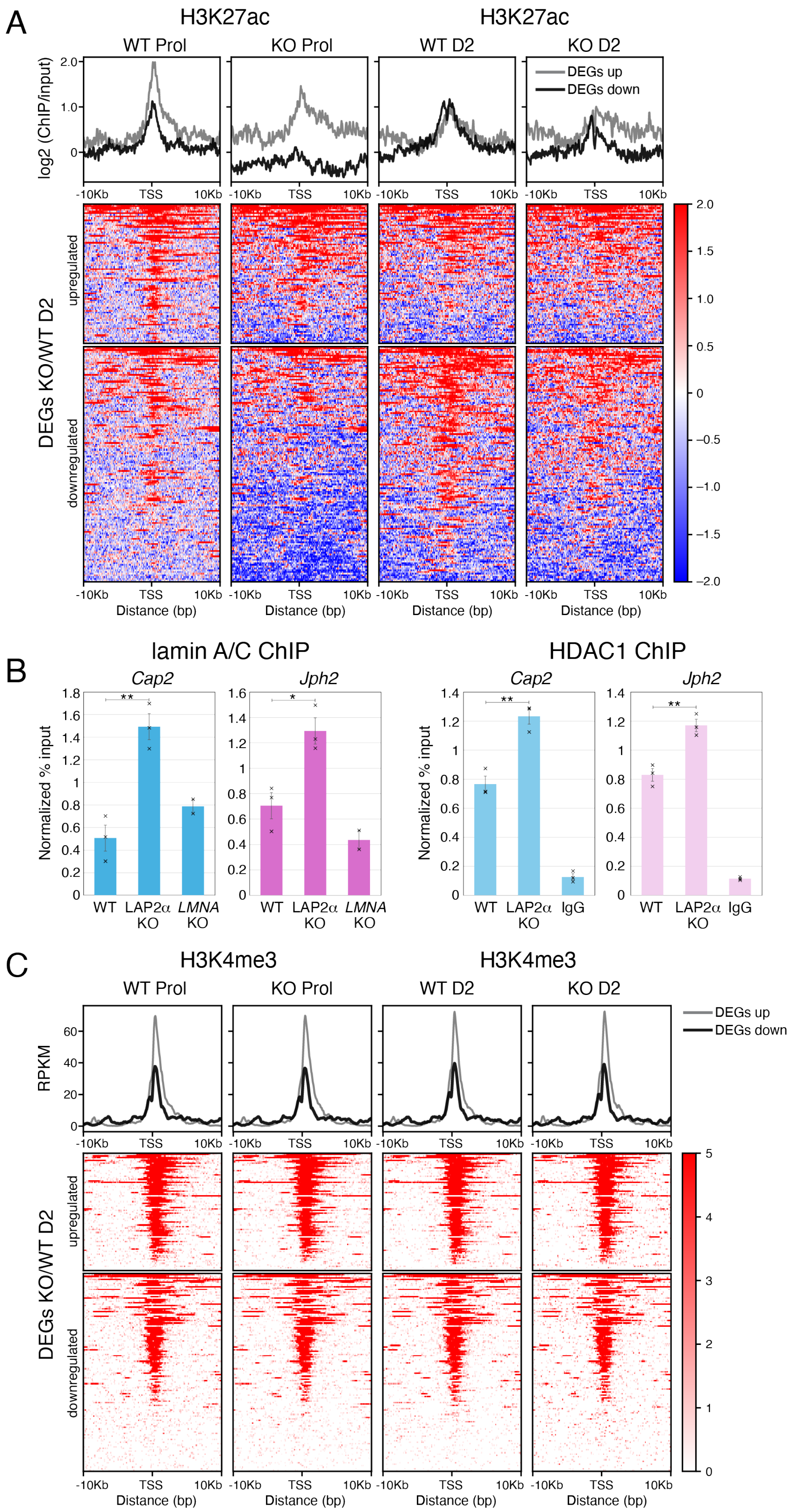
A
H3K27ac
H3K27ac
WT Prol
KO Prol
WT D2
KO D2
DEGs up
DEGs down
log2 (ChIP/input)
DEGs KO/WT D2
upregulated
downregulated
-10Kb
TSS
10Kb
Distance (bp)
B
lamin A/C ChIP
HDAC1 ChIP
Cap2
Jph2
Normalized % input
WT
LAP2α KO
LMNA KO
IgG
C
H3K4me3
H3K4me3
RPKM

Figure S7. **H3K27ac marks are reduced on the transcription start site of deregulated genes in LAP2α knockout cells, whereas H3K4me3 is unaltered. (A)** Heat maps displaying log2 ratio signal (ChIP over input) for H3K27ac in proliferating (Prol) and differentiating (D2) wildtype (WT; panel 1 and 3) and LAP2α knockout myoblasts (KO; panel 2 and 4) on genes that are differentially expressed in LAP2α knockout versus wildtype cells at differentiation day D2 (DEGs KO/WT D2; 215 genes) split into up- and downregulated genes. Graphs on top of heatmaps show mean log2 ratio tracks. **(B)** ChIP-qPCR analysis was performed in WT and LAP2α KO cells using antibodies to lamin A/C (left panel) and HDAC1 (right panel) or unspecific IgG antibodies as a control (IgG). Additionally, *Lmna* KO myoblasts were used as a negative control for lamin A/C ChIP. Precipitated chromatin was analyzed using primers specific to the regulatory region (+/–1kB up- and downstream of TSS) of two genes (*Cap2, Jph2*) that are downregulated in LAP2α KO cells. Data are displayed as batch-normalized average % input (see Materials and Methods for details) ± standard error of 3 biological replicates. Single data points are depicted for each column. Lamin A/C ChIP: **p-value$_{Cap2}$ = 0.0038; *p-value$_{Jph2}$ = 0.0155; HDAC1 ChIP: **p-value$_{Cap2}$ = 0.0036; **p-value$_{Jph2}$ = 0.00497 (two-tailed, two-sample, equal variance student's t-test). **(C)** Heat maps as in (A), but displaying RPKM-normalized H3K4me3 ChIP signal on up- and downregulated DEGs KO/WT D2 in proliferating and D2 wildtype (panel 1 and 3) and LAP2α knockout cells (panel 2 and 4). Graphs on top of heatmaps show mean RPKM signal. TSS: transcription start site.
